# Natural products for enhancing the sensitivity or decreasing the adverse effects of anticancer drugs through regulating the redox balance

**DOI:** 10.1186/s13020-024-00982-2

**Published:** 2024-08-20

**Authors:** Yitian Sun, Qinyi Li, Yufei Huang, Zijing Yang, Guohua Li, Xiaoyu Sun, Xiaoqing Gu, Yunhao Qiao, Qibiao Wu, Tian Xie, Xinbing Sui

**Affiliations:** 1 State Key Laboratory of Quality Research in Chinese Medicines, Faculty of Chinese Medicine, Macau University of Science and Technology, Macau, 999078 China; 2 College of Pharmacy, Hangzhou Normal University, Hangzhou, 311121 Zhejiang China

**Keywords:** Natural products, Cancer, Redox, Sensitization, Detoxification

## Abstract

Redox imbalance is reported to play a pivotal role in tumorigenesis, cancer development, and drug resistance. Severe oxidative damage is a general consequence of cancer cell responses to treatment and may cause cancer cell death or severe adverse effects. To maintain their longevity, cancer cells can rescue redox balance and enter a state of resistance to anticancer drugs. Therefore, targeting redox signalling pathways has emerged as an attractive and prospective strategy for enhancing the efficacy of anticancer drugs and decreasing their adverse effects. Over the past few decades, natural products (NPs) have become an invaluable source for developing new anticancer drugs due to their high efficacy and low toxicity. Increasing evidence has demonstrated that many NPs exhibit remarkable antitumour effects, whether used alone or as adjuvants, and are emerging as effective approaches to enhance sensitivity and decrease the adverse effects of conventional cancer therapies by regulating redox balance. Among them are several novel anticancer drugs based on NPs that have entered clinical trials. In this review, we summarize the synergistic anticancer effects and related redox mechanisms of the combination of NPs with conventional anticancer drugs. We believe that NPs targeting redox regulation will represent promising novel candidates and provide prospects for cancer treatment in the future.

**Graphical Abstract:**

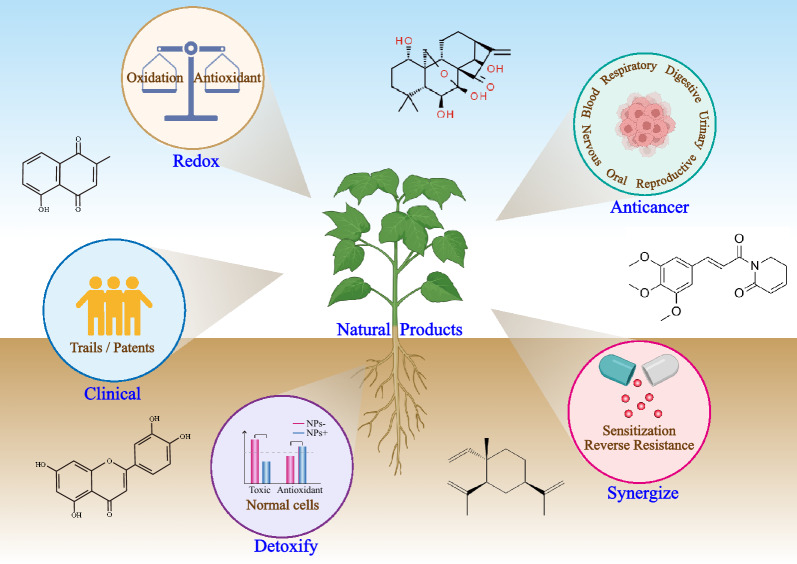

## Highlights

- An abnormal redox microenvironment plays a critical role in the tumorigenesis, development, and drug resistance of cancer.
- NPs can selectively act as pro-oxidants, inducing cytotoxicity in cancer cells, while also serving as antioxidants and protectors in normal cells for detoxification.
- NPs play a dual role in increasing the sensitivity and reversing the drug resistance of cancer cells, primarily through pro-oxidant action and, in some cases, through antioxidant action.
- The heterogeneities in cellular and molecular biology provide the potential basis for the diverse regulation of NPs on the redox state.
- The advances in novel redox-sensitive materials and clinical applications of NPs offer promising prospects.

## Introduction

The term “redox” is derived from the concepts of reduction and oxidation, which are processes that are pivotal for maintaining cellular homeostasis, regulating a wide array of cellular processes, and impacting the overall functionality of cells. The redox balance is dynamically responsive, with relatively reducing steady states and relatively oxidizing steady states, which are maintained by the regulation of oxidation and antioxidant systems [1]. Reactive oxygen and nitrogen species (ROS/RNS) are key players in the regulation of the redox microenvironment. ROS/RNS are a class of highly reactive radicals, ions, or molecules that contain unpaired electrons, primarily consisting of superoxide anion radical (O_2_^• −^), hydroxyl radical (^•^OH), hydrogen peroxide (H_2_O_2_), nitric oxide (NO), and peroxynitrite (ONOO^−^) [2, 3]. Moreover, the antioxidant defence system is activated in living cells to combat the harmful effects of oxidative stress. The reduced glutathione (GSH)/glutathione disulfide (GSSG) redox couple is the predominant intracellular antioxidant that acts as a free radical scavenger and inhibitor of peroxidation [4]. Another critical system responsible for maintaining cellular redox homeostasis is the thioredoxin (Trx)/thioredoxin reductase (TrxR, encoded by TXNRD) dependent system, which protects organisms from oxidative stress and regulates the expression of proteins via transcription factors [5]. These main antioxidant systems require nicotinamide adenine dinucleotide phosphate (NADPH) as an electron donor. In addition, within cells, a series of regulatory mechanisms are involved in redox processes, such as the Keap1/Nrf2 transcription factor, NF-κB signalling, the MAPK pathway, PI3K-Akt cascades, and the AMPK-mTOR axis [6, 7]. These systems complement each other and jointly participate in the complex regulation of intracellular redox homeostasis, particularly in cases involving pathological conditions.

Cancer tumorigenesis and development are extremely complex and involve multistep processes that are highly dependent on the environment, from tissues to patients [8]. Redox levels play a critical role in modulating cell fate. Appropriate redox homeostasis is involved in signal transduction pathways in cells and maintains normal biological functions. However, imbalanced redox causes oxidative damage to important intracellular biomolecules, leading to abnormalities in the structure and function of cells [9]. Oxidative stress is closely associated with cancers and is defined as a state in which the level of ROS overrides the antioxidant defence mechanisms of the cell [10]. Specifically, most cancer cells exhibit more ROS compared with normal cells, indicating higher levels of oxidative stress, which confers advantages for increasing carcinogenesis and development [11]. Moreover, cancer cells can maintain a delicate balance of intracellular ROS levels. The activity of antioxidant systems in drug-resistant cancer cells is significantly increased compared with that in drug-sensitive cells [12]. This self-regulation not only allows cancer cells to adapt to persistent intrinsic oxidative stress and prevents cell death but is also considered an important mechanism of treatment resistance. In addition, abnormal redox reactions in cancer cells may increase their susceptibility to damage by ROS induced by exogenous reagents [13]. To date, various drugs with regulatory effects on ROS levels have been used for cancer treatment. However, the toxicity of these traditional treatments on nontumour cells, which is partially caused by oxidative stress, remains a critical issue. Consequently, targeting redox regulation in cancer cells is expected to represent an effective approach for enhancing sensitivity or reducing adverse effects.

**Fig. 1:**
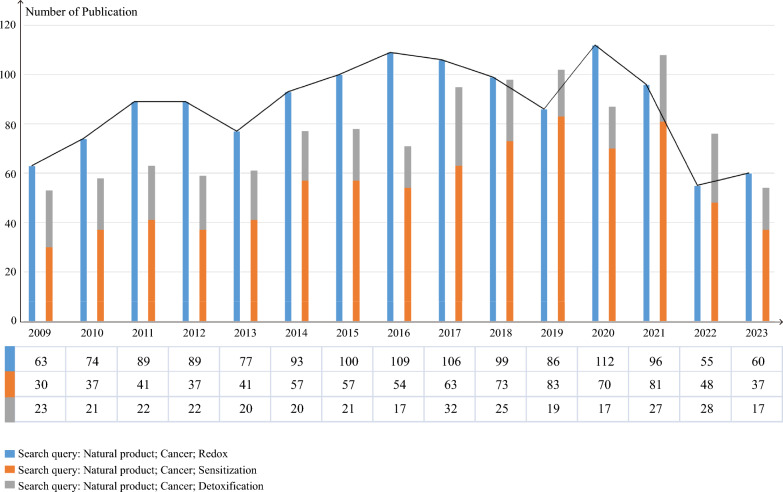
The number of PubMed publications related to NPs, cancers, and redox over the past few years. An increasing number of reports revealed that NPs have served as a cornerstone for cancer treatment involving redox regulation

Natural products (NPs) have various sources in nature and have served as a cornerstone in the search for new cancer therapies because of their diverse biological activities and chemical structures [14] (Fig. 1). Although a series of studies have helped elucidate the anticancer mechanisms of NPs, the findings in this field are still obtained from separate and individual studies and remain controversial. Therefore, systematic and comprehensive retrospective analyses are necessary. Herein, we provide an overview of the mechanisms of sensitization and detoxification in cancer cells by NPs through the regulation of the redox microenvironment. Overall, considering the imbalanced redox in cancer cells, NPs that are useful for redox regulation represent a promising class of drug candidates that may offer potential clinical and patient benefits, suggesting a potential shift in the paradigm of cancer therapy based on NPs.

## Background: association between redox regulation and cancer

**Fig. 2:**
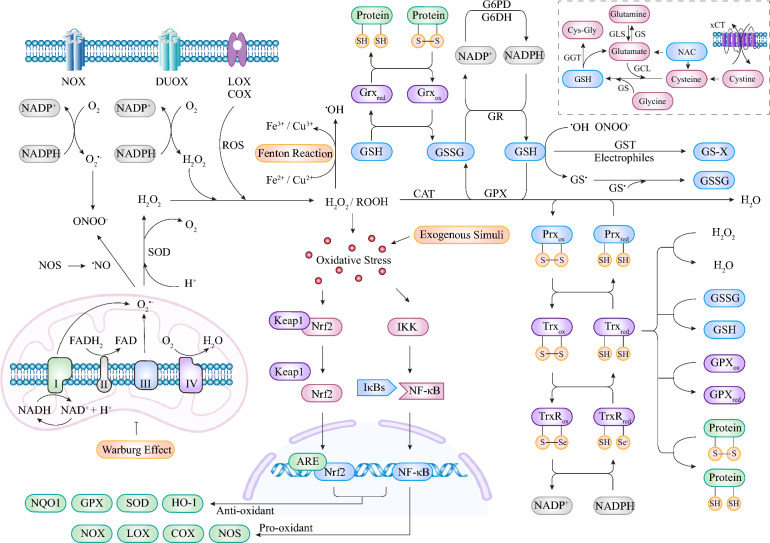
Regulation of the intracellular redox balance. Oxidation processes generate ROS/RNS to serve as key players in the regulation of redox balance and involve electron transport during OXPHOS, oxidoreductase enzymes, the Fenton reaction, and exogenous factor stimulation. The aerobic metabolism of cancer cells also progresses via the Warburg effect, which reduces ROS produced by mitochondrial OXPHOS. Typical reductive equivalents are generated from antioxidant systems, including GSH/GSSG and Trx/TrxR active couples, both of which require the NADP^+^/NADPH system as an electron donor. These substances interact with each other and collectively participate in the complex regulation of intracellular redox homeostasis

The regulation of redox reactions is an ancient topic that has been extensively explored and involves numerous oxidation processes and reduction equivalents (Fig. 2). The intracellular oxidation process involves several biological mechanisms, including the mitochondrial electron transport chain, oxidoreductase enzyme mediation, metal-catalysed oxidation, and exogenous stimulation. Typically, intracellular reductive equivalents are generated from reductive substrates, and the relevant active couples constitute the major antioxidant systems that maintain redox balance and prevent intracellular oxidative damage. Understanding this background can help us discover potential targets and apply them in research on either physiological or pathogenic processes.

### Regulation of oxidative and antioxidant pathways

It has been reported that mitochondria in cancer cells are characterized by ROS overproduction [15]. Mitochondrial redox signalling relies mainly on ROS generated by the electron transport chain via oxidative phosphorylation (OXPHOS), in which significant changes in the potential of electrons are related to the reduction of oxygen [16]. Conversely, the aerobic metabolism of cancer cells also progresses away from mitochondrial oxidative metabolism, known as the Warburg effect, which reduces ROS production by OXPHOS through the upregulation of the glycolysis rate in energy metabolism [17]. In such cancer cells, ROS are also generated through enhanced metabolism caused by abnormal signal transduction and gene activation [18]. Other pro-oxidant enzymes include NADPH oxidase (NOX/DUOX), lipoxygenase (LOX), cyclooxygenase (COX), and nitric oxide synthase (NOS), among others [19]. Among them, NOXs are transmembrane enzymes that are involved in the production of ROS and are overexpressed in multiple malignancies, leading to redox imbalance and tumour progression; thus, NOXs constitute potential targets for cancer treatment [20]. Additionally, the oxidative process can also be mediated by transition metals (such as ferrous and copper ions) through a nonenzymatic process known as the Fenton reaction. Exogenous carcinogens that contribute to the oxidation load include radiotherapy, chemicals, stress, and other toxicants [19].

GSH represents the most abundant low-molecular-weight thiol in cells, and the nucleophilic cysteine-thiol (R‒SH) group is the reactive group of the molecule, mediating its various biological activities. The ratio of GSH (reduced) to its disulfide, GSSG (oxidized), contributes to the redox homeostasis of the cell, and a decrease of the GSH/GSSG ratio serves as a biomarker of oxidative stress [21]. First, because of the cysteine residue, GSH is readily oxidized nonenzymatically to disulfide by electrophilic substances [22]. Second, GSH is extensively used as a cosubstrate by antioxidant enzymes such as glutathione peroxidase (GPX), glutaredoxin (Grx), and certain glutathione S-transferases (GSTs) [23]. In addition, Grx has been shown to promote reversible protein S-glutathionylation using a glutathionyl radical as the proximal donor [24], thereby preventing ROS-induced irreversible protein oxidative damage. GST is a key enzyme that catalyses the conjugation of GSH to a variety of electrophilic substances and the formation of GS-X and plays a critical role in cellular detoxification and signalling [25]. As a widely applied antioxidant, N-acetylcysteine (NAC) affects redox regulation by replenishing GSH, reducing disulfide bonds in proteins or other molecules, or directly scavenging oxidants and is often used as a supplement that may help with various conditions [26].

The Trx/TrxR active couple is overexpressed in various types of cancers [27]. Trx1 and Trx2 are two Trxs ubiquitously expressed in mammalian cells. As a thiol-disulfide reductase, Trx can catalyse the reduction of disulfides (S–S) within oxidized cellular proteins such as peroxiredoxin (Prx, HGNC root symbol PRDX) due to the presence of two cysteines in Trx’s active centre, Cys-Gly-Pro-Cys [28]. Trx also participates in cofactor activities and regulates the expression of many redox-related transcription factors, including nuclear factor kappa B (NF-κB), nuclear factor erythroid 2-related factor 2 (Nrf2), and redox factor-1 (Ref-1) [28]. TrxR is a homodimeric selenocysteine-containing flavoprotein that controls the redox state of Trx. The oxidized Trx can be further regenerated through reduction by TrxR at the expense of electrons provided by NADPH [29]. Three mammalian isoforms of TrxR have been characterized in humans: cytosolic TrxR1, mitochondrial TrxR2, and TrxR3 [30]. As an endogenous negative regulator of Trx, thioredoxin-interacting protein (TXNIP) is also a key redox protein and a potential therapeutic target [31].

Both GSH/GSSG and Trx/TrxR ultimately rely on the reducing power of NADPH, which functions as an important cofactor to provide electrons in antioxidant defence systems [32]. NADPH donates two electrons to reduce GSSG to GSH via GR. TrxR transfers electrons from NADPH to reduce oxidized thioredoxin (Trx-S_2_) to Trx-(SH)_2_, which provides reducing equivalents in the enzymatic removal of H_2_O_2_ and other organic hydroperoxides by Prx [33]. The generated NADP^+^ can primarily be reduced back to NADPH mainly by G6PD in the pentose phosphate pathway in the cytoplasm and by IDH2 and NNT in the mitochondria [34]. The regulation of enzymes involved in the biosynthesis of NADP^+^ and NADPH can influence NADP^+^/NADPH levels and the cellular redox potential [35]. Although NADPH is considered to be the electron source of antioxidant systems, it also acts as a substrate for the ROS-producing NOX family [36]. Overall, cellular redox homeostasis primarily results from a delicate balance between NADPH-dependent antioxidant systems (GSH/GSSG, Trx/TrxR, and Prx) and NADPH-dependent NOX or oxidation processes.

In addition to these critical redox systems, a series of transcription factors and signalling pathways participate in redox regulation within cells. Under normal conditions, Nrf2 is localized to the cytoplasm, where it interacts with Keap1. However, Keap1 becomes oxidized when exposed to oxidative or electrophilic stress, resulting in the synthesis of Nrf2 and its entry into the nucleus to regulate target gene expression [37]. Nrf2 activation is also regulated by the Trx/TrxR system, which acts as a negative regulator of Nrf2 transactivation [38]. Nrf2 has several target genes and regulates the expression of various antioxidants, such as GST, GPX, catalase (CAT), superoxide dismutase (SOD), and haem oxygenase-1 (HO-1) [37]. NF-κB is another redox-sensitive factor that can interact with the Nrf2 pathway. One of its most important influences in regulating redox balance is the increase in antioxidant proteins [39]. However, it is remarkably noteworthy that the regulation of redox reactions by NF-κB may be bidirectional, as it also plays a pro-oxidant role [39]. In addition, multiple signalling pathways can perceive redox changes and mediate downstream effects. As messengers, ROS/RNS-mediated cellular signalling involving the MAPK (ERK/JNK/p38), PI3K-Akt, and PKC signalling pathways, has been well reviewed [40, 41].

### Redox imbalance, carcinogenesis, and drug resistance

Redox imbalance is considered an important stressor that controls tumour cell growth. Most reports on the associations between oxidative stress and carcinogenesis/progression revolve around ROS, which serve as the core factor that influences redox balance [42, 43]. ROS are considered to contribute to both tumorigenesis and development through the following mechanisms: (1) inducing DNA damage through the oxidation of nucleobases [44]; (2) regulating redox-sensitive transcription factors in cancer, including NF-κB, Nrf2, p53, and AP-1 [45]; (3) influencing the expression of oncogenes and tumor-suppressor genes by epigenetic modifications [46]; (4) acting as signalling molecules to drive cellular proliferation via the PI3K/AKT/mTOR and MAPK/ERK mitogenic signalling cascades [47]; and (5) promoting epithelial-to-mesenchymal transition [48]. Cancer cells generally exhibit increased oxidative stress, which promotes tumour initiation, growth, and proliferation; however, excessive ROS is harmful to cells. When ROS accumulation exceeds the tipping point, their carcinogenic effects on proliferation and development are shifted to antitumour effects via the induction of programmed cell death (PCD) [47, 49], including apoptosis, autophagy, necroptosis, pyroptosis, and ferroptosis. To neutralize ubiquitously elevated oxidative stress and maintain favourable redox homeostasis, cancer cells are able to adjust multiple antioxidant enzymes that support tumour progression, resulting in increased levels of GSH/GSSG [50], Trx/TrxR [27], NADPH [32], and other related proteins. The adaptation mechanism of cancer cells not only contributes to their survival under conditions of persistent oxidative stress but also results in resistance to certain anticancer agents [51]. This finding sets the tone for redox-mediated treatment, demonstrating that targeting redox regulation is a potent candidate approach for cancer therapy.

The efficacy of cancer therapies is partly due to the ROS production and consequent induction of oxidative stress in cancer cells. However, cancer cells can develop resistance to oxidative stress provoked by the treatment, which mechanistically involves an increase in antioxidation systems. For example, compared to those in drug-sensitive cells, the ratios of glutamate/glutamine and GSH/GSSG on the membrane of drug-resistant tumour cells are significantly greater (indicating “glutamine addiction”) [52]. Glutaminase (GLS) catalyses glutaminolysis to promote the formation of glutamate, which is important for GSH synthesis. Sorafenib-resistant hepatocellular carcinoma cells exhibit upregulated GLS1 and resistance to oxidative stress. Inhibiting glutamine metabolism sensitizes sorafenib-resistant cells to sorafenib [53]. The Trx/TrxR system is another contributor to cancer resistance, and increased Trx/TrxR expression is associated with cancer cell resistance to various chemotherapeutic agents. For example, Trx confers a growth advantage to pancreatic cancer cells and increases their resistance to cisplatin-induced apoptosis [54]. In malignant melanoma cells, intracellular Trx/TrxR expression together with endogenous TNFα is correlated with resistance to TNFα-induced cytotoxicity [55]. Trx1 is also involved in paclitaxel-induced drug resistance in ovarian cancer cells [56]. In addition, higher levels of NADPH are observed in drug-resistant cancer cells, which are considered to be more resistant to oxidative stress and to help to prevent ROS-mediated damage [53]. Therefore, targeting the antioxidation system that maintains cancer cell adaptation is a candidate approach for overcoming drug resistance. Some compounds have been shown to be effective in this context. For example, Trx inhibitors such as auranofin have entered clinical trials to improve therapeutic sensitivity and may be of significant clinical value [57].

## Discovery: anticancer effects of NPs by regulating the redox balance

**Fig. 3:**
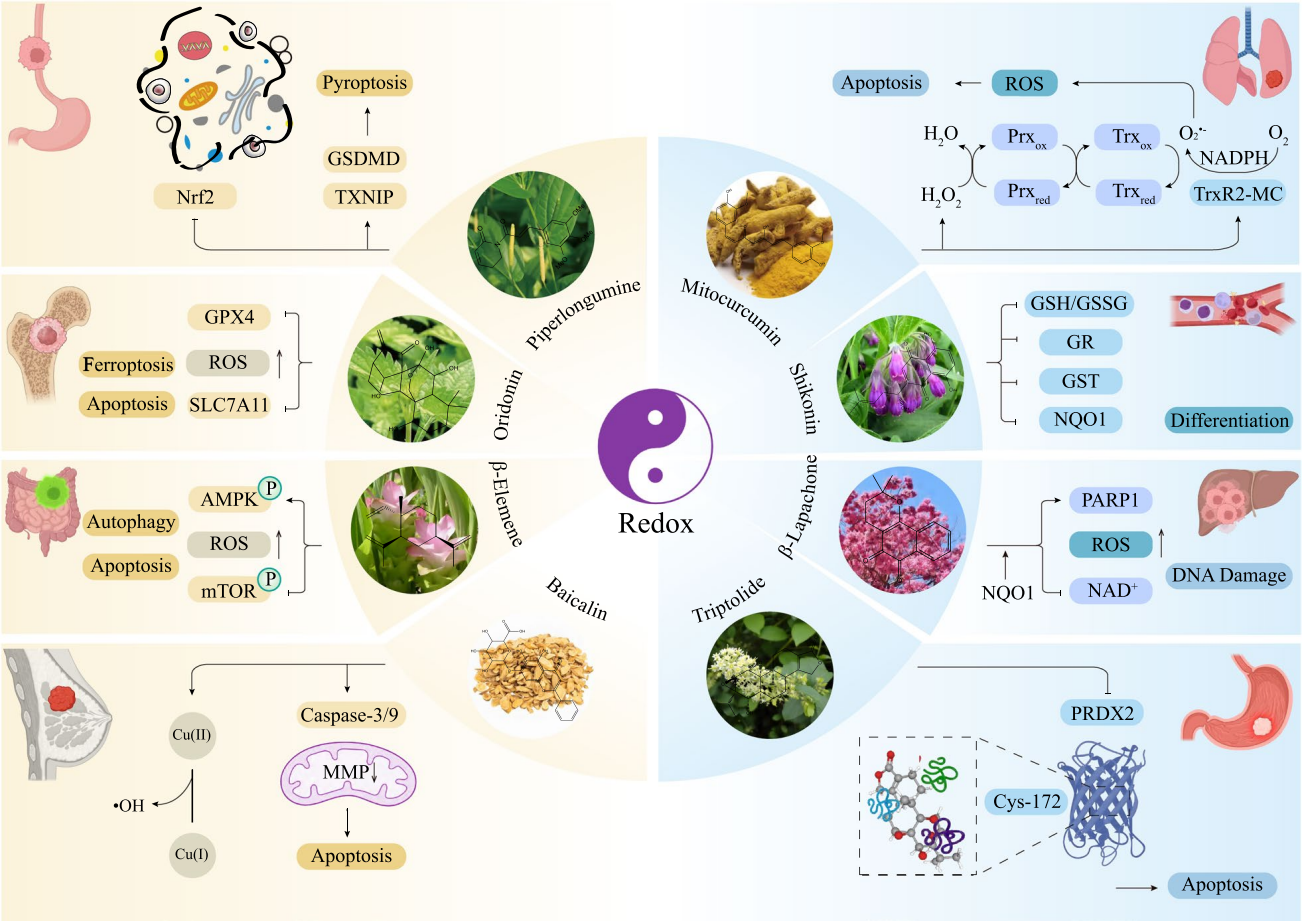
The anticancer activities of NPs in different cancers involve redox regulation. Various types of NPs, including terpenoids, phenolics, flavonoids, quinones, and alkaloids, exert inhibitory effects on multiple cancers by regulating the redox state, primarily by targeting intracellular antioxidant systems or directly promoting ROS generation, thereby inducing PCD

NPs are naturally derived from a wide range of plant sources and have various biological activities; thus, they constitute a cornerstone in the search for new cancer therapies. Here, we provide several typical examples to illustrate different types of NPs and their anticancer mechanisms (Fig. 3), and it is convincing that NPs act as attractive oxidative stress regulators. Given their excellent pharmacological potential, NPs and their derivatives and analogues are gradually being incorporated into therapeutic drug repositories for cancer treatment.

### Terpenoids

Terpenoids constitute the largest class of NPs and have attracted considerable attention as drug libraries. The regulation of the redox state has been well established as a reason for the anticancer effect of terpenoids. For example, andrographolide reacts with the thiol group of GSH, thus increasing the level of intracellular ROS, and the combination of andrographolide and tanshinone IIA has synergistic effects by promoting crosstalk between ROS and p53 [58]. Tanshinone IIA promotes ferroptosis in breast [59] and gastric [60] cancers by inhibiting SLC7A11, which is accompanied by increased ROS. β-Elemene triggers ferroptosis and increases lipid ROS in lung cancer cells, which may be induced by TFEB-mediated GPX4 degradation [61]. Celastrol suppresses tumours by covalently targeting PRDX in both gastric [62] and colorectal [63] cancers. Celastrol triggers apoptosis and autophagy by activating the ROS/JNK pathway, as observed in cases of osteosarcoma [64] and glioma [65]. Artesunate induces autophagy-dependent apoptosis in bladder cancer by activating the ROS/AMPK/mTOR/ULK1 pathway [66]. Artesunate has also been identified as an activator of ROS-dependent ferroptosis in several cancers [67, 68].

One of the anticancer mechanisms of terpenoids is the Michael addition of their α,β-unsaturated carbonyl units to cellular thiols, which can disrupt the redox state [69]. *Rabdosia rubescens* is a perennial herb native to regions of Asia that has been used for hundreds of years in traditional Chinese medicine [70]. Oridonin is one of the most important compounds isolated from *Rabdosia rubescens*. An investigation of the relationship between the structure and anticancer activity of oridonin revealed that the enone system in the D-ring of oridonin is the active anticancer centre [71]. This structure can undergo irreversible Michael addition reactions with nucleophilic groups of receptor amino acids/proteins, which mediate the regulation of redox-related targets by oridonin. As mentioned previously, GSH expression is generally increased in cancer cells. The anticancer effect of oridonin through GSH depletion has been reported in several human cancers, such as esophageal squamous cell carcinoma [72] and epidermoid carcinoma [73]. Supplementation with GSH or the GSH precursor NAC abolished the toxic effect of oridonin, suggesting that oridonin-induced cell death is ROS dependent. Moreover, the Michael effect between oridonin and GSH was greater than that between ponicidin and GSH, which may explain the better anticancer activity of oridonin. Novel dienone analogues with an additional α,β-unsaturated ketone system on the A-ring of oridonin have been prepared and demonstrated efficacy in inducing apoptosis in breast cancer cells [74].

Parthenolide, a natural sesquiterpene lactone isolated from the traditional Chinese medicinal plant *Tanacetum parthenium*, represents another electrophile that can modify biological nucleophilic molecules through Michael addition, and the highly reactive α-exo-methylene γ-butyrolactone (αMgB) group is the structure responsible for this bioactivity. For example, parthenolide increases GSH oxidation in hepatocellular carcinoma cells, and an increase in thiol oxidation results in an overall increase in lipid oxidation levels, increasing the sensitivity of cells to ferroptosis [75]. In colorectal cancer cells, parthenolide rapidly depletes GSH and protein thiols and increases intracellular ROS and endoplasmic reticulum (ER) stress, further inducing apoptosis [76]. Moreover, parthenolide likely follows the same mechanism to bind of the Sec residue of proteins. Skalska et al. identified surface Trx1 as a target of parthenolide and attributed its anti-lymphoma activity to its ability to modify the redox state of cell exofacial thiols [77]. Duan et al. reported that parthenolide selectively targets TrxR1 and TrxR2, and further induced ROS-mediated apoptosis in HeLa cells [78]. In addition, parthenolide selectively activated NOX and suppressed Trx, MnSOD, and CAT in prostate cancer cells [79]. Overall, an important component of parthenolide’s anticancer activity derives from the reaction of its lactone moiety with thiols.

*Tripterygium wilfordii* is a traditional Chinese medicine that was first recorded in the *Compendium of Materia Medica* (Ben Cao Gang Mu). Triptolide is a diterpene triepoxide extracted from *Tripterygium wilfordii* that has strong bioactivities. Previous studies have demonstrated that triptolide inhibits the translocation of Nrf2 from the cytoplasm to the nucleus, as well as the expression of the downstream genes NAD(P)H dehydrogenase [quinone] 1 (NQO1), CAT, and HO-1, thus abrogating Nrf2-mediated defense mechanisms against oxidative stress [80]. The suppression of Nrf2 by triptolide is closely related to its anticancer activity in several cancers, including glioma [81], lung cancer [82], and colorectal cancer [83]. Chen et al. identified PRDX2 as a direct binding target of triptolide, triptolide covalently binds to PRDX2 and inhibits its activity, inducing ROS accumulation and apoptosis in gastric cancer [84]. Triptolide induces ROS production, subsequently triggering PCD in different cancer cells. For example, triptolide induces apoptosis and autophagy in glioma cells by upregulating the ROS/JNK axis [85]. ROS generation and ERK activation are the major mechanisms by which triptolide induces apoptosis in breast cancer cells [86]. In contrast, triptolide was found to attenuate ionizing radiation-induced pulmonary fibrosis by inhibiting the axis of alveolar macrophages-NOXes-ROS-myofibroblasts axis, thereby reducing ROS [87]. This dual action appears to favor the benefits of triptolide under different pathological landscapes.

### Phenolics

Plant tissues are rich in a wide variety of phenolic compounds, such as kaempferol, curcumin, resveratrol, catechins, and epigallocatechin-3-gallate (EGCG). Phenolic compounds are widely used in health care to prevent undesirable oxidation. EGCG is the most active and abundant polyphenol and has attracted considerable attention in cancer therapy because of its antioxidant activity. EGCG inhibits the process of multistage carcinogenesis and metastasis in multiple tumour models by regulating NOS, COX, Nrf2/Keap1, and other genes [88]. In contrast, phenolic compounds with anticancer potency also increase oxidative stress levels and cause cell death. ROS-mediated apoptosis plays a critical role in the anticancer effects of kaempferol in colorectal [89], glioblastoma [90], and pancreatic cancers [91]. Kaempferol is also used as a natural anticancer agent and sensitizer for lung cancer because it inhibits Nrf2 [92]. Resveratrol suppresses Notch1/PTEN/Akt signalling in ovarian cancer cells through ROS generation [93]. The anticancer activities of resveratrol are also related to manipulation of the levels of Nrf2, SOD, and CAT, for example [88]. Overall, the regulation of the redox state via pro-oxidants or antioxidants by phenolics holds significance for cancer therapy.

Curcumin is a main natural polyphenol that was first extracted from *Curcuma* species two centuries ago, and over 100 different clinical trials involving curcumin have been completed [94]. Curcumin increases Trx1 oxidation and subsequent apoptosis in prostate cancer cells [95]. A curcumin analogue (curcuminoid B63) has been shown to induce ROS-mediated paraptosis-like cell death by targeting TrxR1 in gastric cancer cells [96]. Indeed, the curcumin-induced inhibition of TrxR may depend on its Michael acceptor function. Specifically, curcumin has been shown to mediate the covalent modification of the active site cysteine (Cys) 496 and selenocysteine (Sec) 497 residues in TrxR, subsequently destroying TrxR reduction activity, a process that is irreversible and further leads to dramatic pro-oxidant effects, the induction of NOX activity, and the production of ROS [97]. As a reductive metabolite of curcumin, tetrahydrocurcumin lacks α,β-unsaturated ketone moieties and does not exhibit inhibitory activity against TrxR1 [98]. For TrxR2, high-affinity active sites that bind to mitocurcumin (a derivative of curcumin), including the active sites on the E, F, A, and B chains, have been identified. Mitocurcumin also regulates TrxR2 activity to generate NOX-like activity, resulting in increased ROS accumulation and apoptosis in lung cancer cells [99]. In addition, a significant decrease in the GSH/GSSG ratio was observed in lung cancer cells after treatment with curcumin [100] or its analogues [101], which led to ROS-induced apoptosis. NAC partially or completely reversed these effects, suggesting that ROS generation may be the underlying cause of curcumin-induced cell death. Curcumin regulates redox reactions, which are also mediated by metal ions. Recently, curcumin was reported to mobilize endogenous copper ions, form ROS, and further inhibit prostate cancer cells [102]. Mechanistically, curcumin reduces Cu(II) to Cu(I) and leads to the formation of H_2_O_2_, which further reacts with Cu(I) through the Fenton reaction to produce ^•^OH. Interestingly, the binding sites of curcumin to Cu also contribute to its antioxidant properties, indicating that both the antioxidant and pro-oxidant effects of curcuminoids could be attributed to the same structural moieties [103].

Erianin is a traditional Chinese medicine extracted from *Dendrobium chrysotoxum Lindl* and has a reputation as the “gold in medicine”. The anticancer property of erianin is mediated by multiple signalling pathways, such as the MAPK, PI3K/AKT, Wnt/β-catenin, and Nrf2 pathways [104]. Migration, invasion, and tumour angiogenesis have represented emerging anticancer targets of erianin in recent years. Redox imbalance-mediated PCD is involved in the anticancer effects of erianin. Erianin-induced apoptosis and autophagy in osteosarcoma cells are attributed to ROS induction, leading to the activation of the JNK/c-Jun signalling cascade [105]. Zhang et al. suggested that the anticancer activity of erianin in liver cancer cells was highly correlated with the regulation of ROS-mediated apoptosis; moreover, erianin-induced oxidative stress in HepG2 and SMMC-7721 cells may be related to immune function [106]. In KRAS colorectal cancer cells, erianin induces autophagy-dependent ferroptosis [107]. Erianin also induced ferroptosis in lung [108] and bladder [109] cancers, accompanied by ROS accumulation and lipid peroxidation, as well as a decrease in GSH and GPX4.

### Flavonoids

Flavonoids are naturally found in a wide variety of sources, including berries, tea, wine, onions, and citrus fruits. The cytocidal effect that flavonoids possess when used in cancers is attributed primarily to their ability to evoke oxidative stress. For example, wogonin induces growth inhibition and cellular senescence in breast cancer cells by suppressing TXNRD2 by altering histone acetylation at its regulatory region [110]. Similarly, the anticancer activity of myricetin and quercetin in A549 cells may be due to the inhibition of TrxR [111]. Genistein suppresses the proliferation of leukaemia cells by decreasing the cellular redox potential (GSH/GSSG), accompanied by the downregulation of NADP-dependent isocitrate dehydrogenase [112]. Luteolin induces ferroptosis in clear cell renal cell carcinoma by inducing the Fenton reaction, GSH depletion, and lipid peroxidation [113]. Apigenin has been shown to have a cytotoxic effect through increasing the levels of ROS and lipid peroxidation (LPO) and represents a promising candidate for the treatment of cervical cancer [114]. Notably, the modulatory effect of flavonoids on the redox state is not absolute. Another perspective suggests that the role of flavonoids in cancer chemoprevention can be attributed to their capacity to quench ROS, RNS, and other radicals, as rutin acts as a tumour inhibitor and significantly reduces superoxide in HT29 cells [115].

*Scutellaria baicalensis* is a species of flowering plant that has been widely used as a medicine in East Asian regions. Baicalin is extracted from *Scutellaria baicalensis* and is a major representative of flavonoids with anticancer activities in multiple cancers. Baicalin acts as a pro-oxidant, triggering mitochondrial apoptosis in breast cancer cells through the mobilization of intracellular copper and the generation of ROS [116]. Baicalin induces ferroptosis in several cancers, including bladder [117] and gastric [118] cancers, with changes in ROS levels. Recently, baicalin was reported to inhibit the proliferation of diffuse large B-cell lymphoma cells through increasing intracellular ROS levels and GSDMD expression to induce pyroptosis [119]. Overall, baicalin promotes PCD in various cancer cells by increasing oxidative stress.

Quercetin, which bears the chemical name “3,5,7,3′,4′-pentahydroxyflavone”, is found in a wide range of berries. Quercetin enhances cellular lipid peroxidation and promotes ferroptosis in gastric cancer cells by targeting the SLC1A5/Nrf2 pathway and decreasing GSH and xCT [120]. Quercetin exposure leads to oxidative stress and ER stress in glioblastoma, accompanied by ROS generation and dysregulation of SOD [121]. Zhang et al. reported that PIG3 mediates the pro-oxidant activity of four flavonoids (kaempferol, quercetin, apigenin, and luteolin), which play crucial roles in the ROS-induced mitochondrial apoptosis in liver cancer cells [122]. Quercetin inhibits COX2 by binding to subunit A which has peroxidase activity, and the differential sensitivity of colorectal cancer cells to quercetin is attributed to COX2-dependent ROS generation [123].

### Quinones

To date, quinones have become the second largest class of antitumour agents approved for clinical use in the US [124]. The most prominent chemical feature of quinones is their ability to undergo reversible oxidation–reduction and form semiquinone and oxygen radicals. In addition, the majority of the reactions of quinones with the nucleophile GSH can be characterized as reductive Michael additions, which produce GSH-hydroquinone conjugates [125]. Therefore, the cytotoxicity of quinone-containing chemotherapeutics may be mediated by the generation of semiquinone free radicals and oxyradicals [126]. Natural quinones significantly induce ROS-mediated apoptosis in pancreatic cancer cells, as observed with thymoquinone, plumbagin, and juglone [127]. Shikonin is considered a natural inducer of ROS, suppressing tumour growth and activating antitumour immunity through multiple molecular mechanisms [128]. Emodin regulates the ROS-mediated JNK and PI3K/AKT signalling pathways, consequently inducing necroptosis and inhibiting glycolysis in renal cancer cells [129]. The antioxidant GPX4 has been identified as a target of plumbagin, and its inhibition leads to PCD in glioma [130] and liver cancer cells [131]. Aloin is a natural antitumour anthraquinone glycoside. Wang et al. reported that aloin attenuates ROS generation in gastric cancer cells by downregulating NOX2 activation, consequently suppressing the phosphorylation of prosurvival signalling pathways [132].

The *Rhubarb* genus is commonly recognized for its edible and medicinal plants, the underground parts of which provide herbal materials. Rhein is a natural anthraquinone extracted from *Rhubarb.* Rhein has excellent antiproliferative effects on breast cancer cells because it induces the activation of the ROS-mediated NF-κB and p53 signalling pathways [133]. Rhein induces apoptosis in oral cancer cells by inducing ROS accumulation and inhibiting the AKT/mTOR pathway [134]. Rhein also increases the generation of ROS and activates JNK signalling in liver cancer cells [135]. Rhein has consistently demonstrated similar anticancer mechanisms that mainly involve the induction of ROS generation, which supports its promising anticancer activities.

The *Lapacho* tree (*Handroanthus impetiginosus*) is a native medicinal tree from southern Morocco and Mesoamerica. β-Lapachone is a natural naphthoquinone compound that was originally isolated from the *Lapacho* tree. The antitumour mechanism of β-lapachone is associated mainly with redox cycling and the production of ROS catalysed by quinone oxidoreductases. NQO1 metabolizes a two-electron reduction of β-lapachone using NADH and NADPH as electron sources, forming an unstable hydroquinone that rapidly auto-oxidizes and generates ROS, thereby inducing cell death [136]. Considering the characteristics of this natural quinone, β-lapachone has been developed to selectively target cancers with a specific increase in the quinone oxidoreductase NQO1. β-Lapachone-induced NQO1-dependent cell death has been reported in several cancers, including lung [137], breast [138], and liver cancers [139].

### Other categories

NPs are diverse, and other categories, such as saponins, alkaloids, and organic acids, can also regulate intracellular redox processes. Ginseng is recognized as the "king of herbs", a plant whose roots have high medicinal value; ginsenosides are the main pharmaceutically bioactive components in ginseng. It has been reported that the anticancer effects of ginsenosides are associated with ROS regulation. In breast cancer cells, for example, ginsenoside Rh1 induces apoptosis and autophagy, which are related to the ROS-mediated Akt pathway [140]. Ginsenoside Rh2 induces increased ROS levels and activates the NF-κB pathway in colorectal cancer cells; it also induces paraptosis, a form of PCD that is characterized by cytoplasmic vacuolization [141]. *Piper longum* L. is a naturally existing edible and medicinal plant that grows as a perennial shrub or as an herbaceous vine. It is native to the Indo-Malaya region and is widely used in traditional medicine [142]. Piperlongumine is a biologically active alkaloid isolated from *Piper longum* L. that has anti-inflammatory, anticancer, antimicrobial, and immunomodulatory properties [143]. Piperlongumine inhibits esophageal carcinoma cells by inhibiting Nrf2 and promoting ROS-TXNIP-NLRP3-mediated pyroptosis [144]. Piperlongumine directly targets the Sec498 residue of TrxR1 to inhibit gastric cancer cells [145]. Additionally, it selectively induces ROS accumulation and cell death in cancer cells but not in normal cells. For example, piperlongumine reduces GSH and induces ROS accumulation in glioblastoma multiforme cells, which further activates the JNK and p38 pathways [146]. Piperlongumine increases the intracellular ROS levels sufficiently to cause lethal oxidative stress in breast cancer cells by inhibiting the antioxidant enzymes CAT, Trx1, and Prx2 [147]. Indeed, it has been widely reported that NPs exhibit significant anticancer effects in multiple cancers by regulating the redox microenvironment. Here, we summarize the related mechanisms that involve genes, proteins, transcription factors, signalling pathways, and PCD, with more detailed content presented in Table 1.

**Table 1:** Anticancer mechanisms of representative NPs involving redox regulation in cancers

NPs	Classification	Plant Sources	Cancer Types	Cells	Mechanisms	PCDs	Ref
Tanshinone IIA	Diterpenoid	Salvia miltiorrhiza	Breast	MCF-7, T47D	GSH↓, SLC7A11↓, MDA↑, ROS↑	Ferroptosis	[59]
Tanshinone IIA	Diterpenoid	Salvia miltiorrhiza	Gastric	BGC-823, NCI-H87	SLC7A11↓, ROS↑	Ferroptosis	[60]
β-Elemene	Sesquiterpene	Curcumae Rhizoma	Lung	A549	GPX4↓, ROS↑, LOOH↑, Fe^2+^↑	Ferroptosis	[61]
β-Elemene	Sesquiterpene	Curcumae Rhizoma	Lung	A549	GSH↓, GSSG↑, SLC7A11↓, ROS↑	Apoptosis	[233]
Celastrol	Triterpenoid	Tripterygium wilfordii	Gastric	SGC-7901	PRDX2↓, ROS↑, ER stress↑	Apoptosis	[62]
Celastrol	Triterpenoid	Tripterygium wilfordii	Colorectal	HCT116, SW620	PRDX1↓, ROS↑	Apoptosis	[63]
Artesunate	Sesquiterpene lactone	Artemisia annua	Bladder	EJ, T24	ROS↑, AMPK/mTOR/ULK1↑	Apoptosis	[66]
Artesunate	Sesquiterpene lactone	Artemisia annua	Colorectal	SW480, HCT116	ROS↑, ER stress↑, Ca^2+^↑	Autophagy	[234]
Oridonin	Diterpenoid	Rabdosia rubescens	Esophageal	TE1, EC109	GSH↓, ROS↑, SLC7A11↑	Apoptosis	[72]
Oridonin	Diterpenoid	Rabdosia rubescens	Osteosarcoma	143B, U2OS	GPX4↓, SLC7A11↓, ROS↑, Fe^2+^↑	Apoptosis, Ferroptosis	[235]
Parthenolide	Sesquiterpene lactone	Tanacetum parthenium	Colorectal	COLO 205	GSH↓, ROS↑	Apoptosis	[76]
Parthenolide	Sesquiterpene lactone	Tanacetum parthenium	Breast	MDA-MB231	NOX↑, GSH↓, ROS↑, NF-κB↓	Autophagy, Necrosis	[236]
Triptolide	Diterpenoid	Tripterygium wilfordii	Glioblastoma	BTIC TS603	Nrf2↓, GSH↓, ROS↑, LPO↑	Apoptosis	[81]
Triptolide	Diterpenoid	Tripterygium wilfordii	Gastric	AGS, IM95	PRDX2↓, ROS↑, ER stress↑	Apoptosis	[84]
Alantolactone	Sesquiterpene lactone	Inula helenium	Cervical	HeLa	TrxR↓, Trx↓, ROS↑	Apoptosis	[237]
Andrographolide	Diterpene lactone	Andrographis paniculata	Lymphoma	Ramos, Granta, HF-1, SUDHL4	GSH↓, ROS↑	Apoptosis	[238]
Brusatol	Triterpene lactone	Brucea javanica	Lung	PC9	Nrf2↓, HO-1↓, GSH↓, ROS↑	Apoptosis	[239]
Ursolic Acid	Triterpenoids	Rosmarinus officinalis	Esophageal	TE-8/12	AKT/mTOR↓, ROS↑	Autophagy	[240]
EGCG	Polyphenol	Green tea	Colorectal	HT29	Nrf2↑, UGT1A↑, UGT1A8↑	/	[88]
Kaempferol	Polyphenol	Kaempferol galanga L	Pancreatic	PANC-1, Mia PaCa-2	TGM2↓, ROS↑, Akt/mTOR↓	Apoptosis	[91]
Kaempferol	Polyphenol	Kaempferol galanga L	Lung	A549, NCIH460	Nrf2↓, NQO1↓, HO-1↓, GST↓	Apoptosis	[92]
Resveratrol	Polyphenol	Berries	Ovarian	A2780, SKOV3	ROS↑, Notch1↓, p-PTEN↑, p-Akt↓	/	[93]
Resveratrol	Polyphenol	Berries	Prostate	PC3, DU145	Nrf2↑, HO-1↑, GSH↑, GPX↑, ROS↓	/	[241]
Curcumin	Polyphenol	Curcuma longa	Prostate	LNCaP, PC-3	Trx1↓, O_2_^• −^↑, H_2_O_2_↑, ROS↑	Apoptosis	[95]
Curcuminoid B63	Polyphenol	Curcuma longa	Gastric	SGC-7901	TrxR1↓, ROS↑, MAPK↑	Paraptosis	[96]
Mitocurcumin	Polyphenol	Curcuma longa	Lung	A549	TrxR2↓, ROS↑, GSH↓	Apoptosis	[99]
Erianin	Polyphenol	Dendrobium chrysotoxum Lindl	Lung	H460, H1299	GSH↓, GPX4↓, Ca^2+^↑, ROS↑, MDA↑	Ferroptosis	[108]
Erianin	Polyphenol	Dendrobium chrysotoxum Lindl	Bladder	RT4, KU-19–19	Nrf2↓, GSH↓, ROS↑, MDA↑	Ferroptosis	[109]
Honokiol	Polyphenol	Magnolia officinalis	Thyroid	KMH-2, ASH-3	ROS↑	/	[242]
Honokiol	Polyphenol	Magnolia officinalis	Neuroblastoma	Neuro-2a	GPR78↑, ER stress↑, ROS↑	Autophagy	[243]
Wogonin	Flavonoid	Scutellaria baicalensis	Breast	MDA-MB-231	TXNRD2↓, ROS↑, DNA damage↑	/	[110]
Baicalin	Flavonoid	Scutellaria baicalensis	Bladder	5637, KU19-19	FTH1↓, ROS↑	Ferroptosis	[117]
Baicalin	Flavonoid	Scutellaria baicalensis	Lymphoma	DB	ROS↑, N-GSDME↑, N-GSDMD↑	Pyroptosis	[119]
Myricetin	Flavonoid	Bayberry	Lung	A549	TrxR↓	/	[111]
Genistein	Isoflavones	Genista tinctoria L	Leukemia	HL-60	GSH/GSSG↓, cICDH↓, ROS↑	Apoptosis	[112]
Luteolin	Flavonoids	Reseda luteola	Renal	786-O, OS-RC-2	HO-1↑, Lip↑, Fe^2+^↑, GSH↓	Ferroptosis	[113]
Apigenin	Flavonoid	Celery	Cervical	HeLa, CaSki, C33A	H_2_O_2_↑, ROS↑, LPO↑	Apoptosis	[114]
Rutin	Flavonoid	Ruta chalepensis L	Lung, Colorectal	A549, HT29	O_2_^• −^↓, ROS↓	/	[115]
Quercetin	Flavonol	Quercetum	Gastric	AGS	SLC1A5↓, Nrf2↓, xCT/GPX4↓, ROS↑	Ferroptosis	[120]
Quercetin	Flavonol	Quercetum	Liver	HepG2	PIG3↑, ROS↑, MMP↓	Apoptosis	[122]
Shikonin	Naphthoquinone	Lithospermum erythrorhizon	Lung	SBC-2, H69	ATF3↑, ROS↑, GSH↓	Ferroptosis	[244]
Shikonin	Naphthoquinone	Lithospermum erythrorhizon	Renal	ACHN, Caki-1	ROS↑, LC3B↑, p62↑	Autophagy	[245]
Emodin	Anthraquinone	Rhubarb	Renal	786-O, OS-RC-2	ROS↑, JNK↑, GLUT↓	Necroptosis	[129]
Plumbagin	Naphthoquinone	Plumabago zeylanica L	Glioma	U251	NQO1↑, xCT↓, GPX4↓, GSH↓, MDA↑	Ferroptosis	[130]
Plumbagin	Naphthoquinone	Plumabago zeylanica L	Liver	HepG2, Hep3B	DUB↓, GPX4↓, ROS↑	Apoptosis	[131]
Aloin	Anthraquinone	Aloe vera	Gastric	HGC-27, BGC-823	NOX2↓, ROS↓, AKT/mTOR↓	/	[132]
Rhein	Anthraquinone	Rhubarb	Breast	MCF-7	ROS↑, NF-κB↑, p53↑	Apoptosis	[133]
Rhein	Anthraquinone	Rhubarb	Oral	Ca9-22, YD-10B	ROS↑, JNK↑, AKT/mTOR↓	Apoptosis	[134]
β-Lapachone	Naphthoquinone	Lapacho tree	Lung	H596, A549	ROS↑, PARP-1↑	Apoptosis	[137]
β-Lapachone	Naphthoquinone	Lapacho tree	Breast	MCF-7	H_2_O_2_↑, ROS↑, PARP-1↑	Necrosis	[138]
Juglone	Naphthoquinone	Juglans mandshurica	Liver	HepG2	ROS↑, MAPK↑	Apoptosis	[246]
Thymoquinone	Benzoquinones	Nigella sativa	Bladder	5637, T24	ROS↑, miR-877-5p↑, PD-L1↓	Apoptosis	[247]
Ginsenoside Rh2	Saponin	Ginseng	Colorectal	HCT116, SW480	ROS↑, NF-κB↑	Paraptosis	[141]
Gypenoside L	Saponin	G. pentaphyllum	Liver	HepG2	ROS↑, ER stress↑, Ca^2+^↑	Vacuolation Death	[248]
Piperlongumine	Alkaloid	Piper longum L	Esophageal	KYSE-30	ROS↑, TXNIP↑, Nrf2↓, GSDMD↑	Pyroptosis	[144]
Piperlongumine	Alkaloid	Piper longum L	Gastric	SGC-7901	TrxR1↓, ROS↑	Apoptosis	[145]
Sanguinarine	Alkaloid	Sanguinaria canadensis	Lymphoma	BC1, BC3	ROS↑, DR5↑, Caspase8↑	Apoptosis	[249]
Chelerythrine	Alkaloid	Chelidonium majus L	Gastric	NCI-N87	TXNRD1↓, ROS↑, ER stress↑	Necroptosis	[250]
Berberine	Alkaloid	Rhizoma coptidis	Colorectal	SW620	ROS↑, JNK/p38↑	Apoptosis	[251]
Ferulic Acid	Organic acids	Ferula foetida	Esophageal	EC-1, TE-4	GSH↓, GPX4↓, SOD↓, ROS↑, MDA↑	Ferroptosis	[252]
Gallic Acid	Organic acids	Gallnut	Pancreatic	PANC-1, MIA PaCa-2	ROS↑, ER stress↑, p38↑	Apoptosis	[253]

## Exploration: NPs enhance sensitivity and overcome resistance by regulating the redox balance

**Fig. 4:**
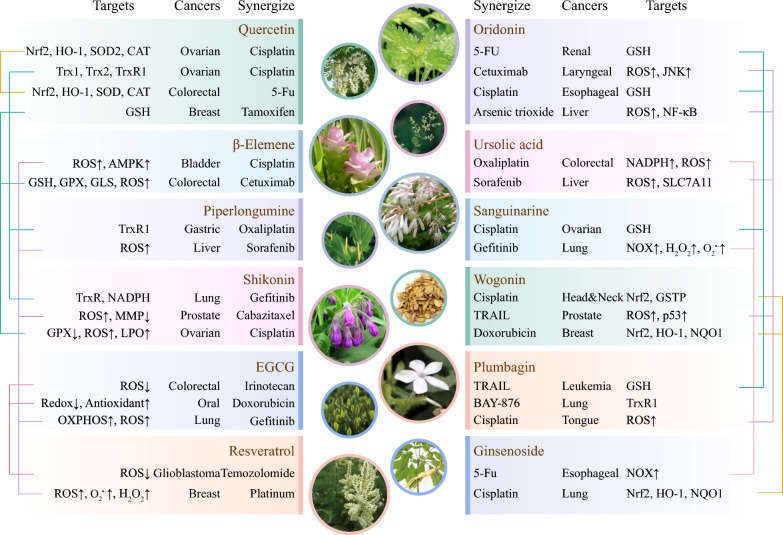
The synergistic mechanisms of the use of NPs as redox-regulating adjuvants in cancers. The combination of NPs with traditional therapies effectively alters the levels of ROS and redox-related targets in various cancer cells

The insensitivity and resistance that evolve in cells during treatment have become increasingly prominent issues affecting therapeutic efficacy. Given the excellent anticancer effects of NPs targeting redox processes, there is increasing interest in combining NPs with conventional therapies to enhance sensitivity or reverse resistance. In recent years, broad reports have provided evidence that NPs are attractive redox-regulating adjuvants for cancer treatment (Fig. 4).

### Targeting the GSH/GSSG system: NPs with Michael acceptor functionality

**Fig. 5:**
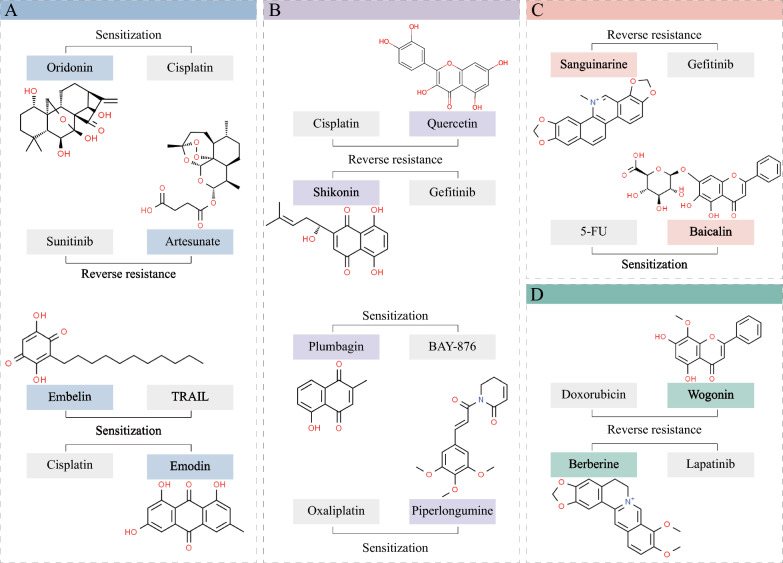
Representative combinations of NPs and chemotherapeutic agents that enhance sensitivity or reverse drug resistance. **A** NPs with Michael acceptor functionality represent potential sensitizers for cancer treatment by targeting GSH. **B** The presence of electrophilic centres may support the activities of NPs for enhancing sensitivity and reversing resistance. **C** Regulating the NADP^+^/NADPH system and increasing the levels of NOXs in cancer cells. **D** NPs targeting Nrf2 or other redox indicators to reverse drug resistance

As mentioned previously, the anticancer effect of oridonin, which targets GSH through the Michael addition reaction, has been reported. Therefore, targeting the GSH/GSSG system may represent an advantageous approach to enhancing oridonin sensitivity and overcoming drug resistance in cancer cells. Platinum compounds are among the most widely used chemotherapy drugs for various types of cancers; however, multidrug resistance is the main barrier preventing their application. It has been revealed that enhanced drug detoxification by GSH or GSH-related moieties is a major mechanism of platinum resistance [148, 149]. Yang et al. reported the synergistic anticancer effects of oridonin and cisplatin against esophageal squamous carcinoma cells, which are likely driven by the inhibition of GSH generation and increased ROS generation, ultimately inducing apoptosis [150]. Oridonin has also been shown to enhance the cytotoxic effect of 5-fluorouracil (5-FU) on renal cancer cells via GSH depletion and ROS formation, further leading to intracellular apoptosis and necroptosis, which are associated with the activation of MAPK and other pathways [151]; moreover, supplementation with GSH or NAC abolished this toxic effect of oridonin, supporting the synergistic effect of GSH/ROS. Tumour necrosis factor-related apoptosis-inducing ligand (TRAIL) is a promising anticancer agent that induces cytotoxicity triggered by interactions with death receptors in cancer cells. Plumbagin is a bicyclic naphthoquinone produced in the roots of *Plumabago zeylanica* L., and its cytotoxic properties are related to its quinone core. Plumbagin is considered to be a potent electrophile that reacts with the thiol group of GSH [152]. Plumbagin enhances TRAIL-induced apoptosis of human leukaemia cells by depleting GSH and increasing the ROS-mediated DRs of TRAIL, exhibiting synergistic effects with TRAIL treatment [153]. Similarly, embelin is a naturally occurring benzoquinone compound that enhances TRAIL sensitivity by suppressing critical antioxidants and increasing ROS accumulation through the consumption of GSH and the downregulation of SOD1 in inflammatory breast cancer cells [154]. Emodin is a natural anthraquinone derivative found in *Rhubarb* that has significant pharmacological effects and clinical applications. Emodin enhances the chemosensitivity of bladder cancer cells to cisplatin by depleting GSH, decreasing GSH-cisplatin conjugates, and elevating ROS levels [155]. Quercetin is another quinone with Michael acceptor properties. It has been reported that GSH levels decrease when breast cancer cells are treated with high concentrations of quercetin, and quercetin combined with tamoxifen synergistically inhibits cell viability [156]. Overall, these observations suggest that compounds with Michael acceptor functionalities are potential sensitizers for cancer treatment [69] (Fig. 5A).

### Targeting Trx/TrxR: a classic pathway for enhancing sensitivity and reversing resistance

High Trx/TrxR levels are important components of the resistant phenotype in various cancers [157–159]. The Sec residue of TrxR seems to be a potent binding site for several NPs. As mentioned before, piperlongumine binds or inhibits the activity of antioxidant enzymes, including Trx1, TrxR1, and PRDX2, in cancer cells [145, 147]. Mechanistically, the α,β-unsaturated ketone moiety in piperlongumine, which serves as a Michael addition acceptor, can bind the C-terminal Sec residue of TrxR [160]. Recently, Zhang et al. reported that the combination of piperlongumine and oxaliplatin significantly inhibited TrxR1 activity in gastric cancer cells, leading to increased ROS production and oxidative stress, suggesting that piperlongumine synergistically potentiates the antitumour effect of oxaliplatin both in vitro and in vivo [161]. Shikonin is a natural naphthoquinone that modifies the Sec498 residue of TrxR1 to fully inhibit its antioxidant activity [162]. Shikonin targets TrxR and induces oxidative stress to promote apoptosis in gefitinib-resistant non-small cell lung cancer cells, with decreases in NADPH and the GSH/GSSG ratio [163]. Similarly, the combination of plumbagin and BAY-876 (a GLUT1 inhibitor) showed synergistic cytotoxicity in lung cancer cells by inhibiting TrxR and ROS generation. Specifically, plumbagin contains an α,β-unsaturated carbonyl functionality that serves as a Michael acceptor, modifies the Sec498 site of TrxR1, and promotes ROS production [164]. Quercetin pretreatment has been shown to act as a pro-oxidant in cisplatin-resistant ovarian adenocarcinoma cells, inducing ROS production by effectively reducing the levels of the Trx/TrxR antioxidant system (Trx1, Trx2, and TrxR1) and downregulating the mTOR/STAT3 pathways, which synergistically potentiate cisplatin cytotoxicity [165]. Zhu et al. synthesized a derivative of oleanolic acid, olean-28,13b-olide 2, which reversed the cisplatin resistance of lung cancer cells by inhibiting TrxR, regulating other pathways, and enhancing cisplatin-induced ROS accumulation [166]. Considering that the inhibition of TrxR by electrophiles usually occurs through Michael addition, the electrophilic centres in NPs likely support their anticancer activity and synergistic effects by targeting the Trx/TrxR system [167] (Fig. 5B).

### Targeting NADP^+^/NADPH: increased expression of the pro-oxidant enzyme NOX

Currently, several NPs have been shown to enhance the sensitivity or reverse the resistance of cancer cells by regulating NADPH or NOXs **(**Fig. 5C**)**. Epidermal growth factor receptor (EGFR) tyrosine kinase inhibitor (TKI) resistance is caused by an additional T790M mutation in EGFR. Sanguinarine is a naturally derived isoquinoline alkaloid from *Sanguinaria canadensis* with various bioactivities, including anti-inflammatory, anticancer, and neuroprotective effects [168]. In gefitinib-resistant non-small cell lung cancer cells, sanguinarine-mediated M790 oxidation occurs through the upregulation of NOX3 and subsequent H_2_O_2_ production. NOX3 oxidizes NADPH, causing severe NADPH depletion and an increase in the NADP^+^/NADPH ratio, ultimately triggering EGFR overoxidation and apoptosis [169]. The ginsenoside Ro-triggered accumulation of superoxides inhibits autophagy and sensitizes esophageal cancer cells to 5-FU-mediated cell death. As important sources of superoxides, both NOX2 and NCF1 were shown to be upregulated by ginsenoside Ro treatment in a dose-dependent manner, contributing to the synergistic anticancer effect of ginsenoside Ro [170]. Baicalin enhances the efficacy of 5-FU in gastric cancer cells by inducing ferroptosis. In this case, alterations occurred within the cellular redox microenvironment, marked by elevated cellular oxidation, increased levels of the oxidase NOX1, and decreased expression of the antioxidant enzyme GPX4 [118]. In addition to forming the antioxidant couple, another role of NADPH is as a substrate for NOX to promote the production of ROS. Therefore, the NADPH concentration could also be increased by NP treatment in certain cases. For example, combination treatment with ursolic acid and oxaliplatin increased NADPH levels, a resource for ROS production, in colorectal cancer cells. Elevated ROS levels markedly reduce the expression of drug resistance genes, including permeability glycoprotein, multidrug resistance-associated protein, and breast cancer resistance protein [171]. In contrast, NPs act as sensitizers by downregulating NOX levels in certain cases. It has been reported that emodin combined with cisplatin downregulates the expression of NOX, along with a decrease in ROS, which is considered to inhibit the expression of drug resistance genes and enhance chemosensitivity in endometrial cancer cells [172].

### Targeting the Nrf2/HO-1 axis and other redox indicators: a potent pathway for reversing drug resistance

High Nrf2 expression is associated with the resistance of several cancers to chemotherapy [173]. The inhibition of Nrf2 by wogonin contributes to its ability to overcome drug resistance in multiple cancers, including liver cancer [174], breast cancer [175], and head and neck cancers [176]. For example, wogonin inhibits drug detoxification by downregulating Nrf2 and its effector GSTP1, thus reversing cisplatin resistance in HN4-cisR and HN9-cisR cells. Suppression of Nrf2/HO-1 by wogonin reverses doxorubicin resistance in MCF-7/DOX cells. In cisplatin-resistant non-small cell lung cancer cells, ginsenoside Rd was found to restore sensitivity to chemotherapy drugs by suppressing Nrf2, which was accompanied by a reduction in the expression of the downstream genes NQO1 and HO-1, as well as a decrease in the levels of the drug resistance genes MDR1 and MRP1 [177]. Berberine resensitizes breast cancer cells to lapatinib by suppressing the transcriptional activation of Nrf2 and increasing ROS levels [178]. Quercetin suppresses the expression of critical antioxidant enzymes, such as Nrf2/HO-1, GPX, SOD, and CAT, in both 5-FU-resistant colorectal cancer cells [179] and cisplatin-resistant ovarian cancer cells [180]. These mechanisms are considered to contribute to overcoming the resistance of colorectal cancer cells to quercetin. Parthenolide prevents the resistance of breast cancer cells to doxorubicin and mitoxantrone through the downregulation of Nrf2, CAT, and MnSOD [181]. NF-κB is not only associated with redox regulation but is also involved in the development of drug resistance in ovarian cancer cells with increased activity. Triptolide increases cellular ROS production by inhibiting complex I of the mitochondrial respiratory chain, and ROS further mediate the inactivation of the NF-κB survival pathway and cell apoptosis, thus reversing cisplatin resistance in ovarian cancer [182]. Overall, targeting these antioxidant indicators is a potent pathway for reversing the drug resistance of multiple cancers (Fig. 5D).

### The regulation of ROS: a bidirectional regulatory effect

**Fig. 6:**
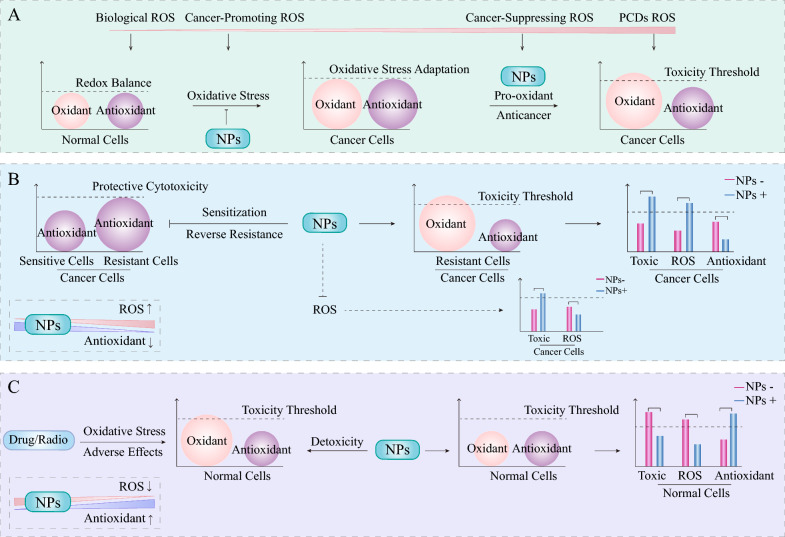
NPs play a dual role in the regulation of redox reactions in different cases. **A** Cancer cells typically exhibit abnormal redox states characterized by elevated levels of oxidative stress and increased antioxidant systems, which contribute to "self-adaptation" and resistance to treatment toxicity. **B** NPs typically act selectively as pro-oxidants in cancer cells to increase sensitivity and reverse drug resistance; however, they can exert synergistic effects by inhibiting oxidative stress. **C** NPs decrease the adverse effects of cancer therapy in normal cells by serving as antioxidants and protectors

ROS are important regulatory factors that determine cell survival and death. It is reasonable to speculate that the signalling pathways discussed earlier may lead to changes in the levels of intracellular ROS, which act as signalling molecules and mediate PCD in cancer cells, consequently supporting the anticancer properties of NPs. Several studies have reported the impact of NPs on tumour ROS; more importantly, future research is necessary to explore the underlying pathways and specific targets involved in these studies. Although most of the mechanisms reviewed above suggest that NPs generally positively regulate ROS in cancers, the bidirectional modulation of the redox microenvironment by NPs cannot be ignored (Fig. 6A, B). For example, quercetin has been shown to enhance the antitumour activity of doxorubicin combined with cyclophosphamide in breast cancer cells by inhibiting ROS accumulation and the ERK1/2 pathway [183]. A reduction in ROS has also been reported as the mechanism by which quercetin overcomes 5-FU resistance in colorectal cancer cells [179]. The regulation of ROS by quercetin may depend on the drug dosage, given that tamoxifen-induced ROS generation was found to be suppressed by low-concentration quercetin, whereas high concentrations of quercetin synergized with tamoxifen to increase the production of ROS [156].

Resveratrol is a phenolic compound that is widely present in various dietary compounds, such as mulberries, peanuts, blueberries, and grapes. It has been reported that resveratrol synergizes with 5-FU to combat colorectal cancer, increasing the ROS levels and decreasing the CAT and GPX levels [184]. The combination of resveratrol and platinum (IV) complex induces increased levels of O_2_^• −^, H_2_O_2_, and NO_2_^−^, further amplifying the pro-oxidative effects of the platinum (IV) complex on both breast adenocarcinoma and choriocarcinoma cells [185]. Resveratrol enhances the antitumour effects of temozolomide in glioblastoma cells via the ROS-dependent AMPK-TSC-mTOR signalling pathway [186]. However, the synergistic effect also likely depends on the antioxidant properties of resveratrol. Resveratrol sensitizes glioma cells to temozolomide-induced cell death synergistically through the downregulation of protective autophagy, leading to a decrease in ROS [187]. EGCG is another major form of dietary polyphenol, and its combined use with EGFR-TKIs has been shown to significantly reverse the Warburg effect, leading to increased OXPHOS and ROS and overcoming drug resistance in non-small cell lung cancer [188]. Wei et al. also showed that EGCG can sensitize renal cell carcinoma cells to TRAIL-induced apoptosis by downregulating c-FLIP via a ROS-dependent pathway [189]. In contrast, EGCG in conjunction with irinotecan has been shown to suppress the production of ROS in colorectal cancer, where the altered redox homeostasis, along with the regulation of GRP78, collectively enhances the chemosensitivity of colorectal cancer cells to irinotecan [190]. The antioxidant properties of EGCG also contribute to reversing multidrug resistance in oral cancer [191]. Overall, NPs enhance cancer sensitivity or reverse resistance by modulating redox balance, a fact that has been well characterized **(**Table 2**)**.

**Table 2:** Representative NPs enhance sensitivity or reverse drug resistance in cancers by regulating redox balance

NPs	Plant Sources	Drugs	Cancer Types	Cells	Mechanisms	Synergistic	Ref
Oridonin	Rabdosia rubescens	Cisplatin	Esophageal	KYSE30	GSH↓, ROS↑, DNA damage ↑	Sensitization	[150]
Oridonin	Rabdosia rubescens	5-FU	Renal	786-O	GSH↓, ROS↑, MAPK↑	Sensitization	[151]
Oridonin	Rabdosia rubescens	Arsenic trioxide	Liver	Bel7402	ROS↑, NF-κB↓, MMP↓, p-p38↑, p-ERK1↑, p-JNK↑	Sensitization	[254]
Oridonin	Rabdosia rubescens	Cetuximab	Laryngeal	HEp-2, Tu212	ROS↑, JNK↑	Sensitization	[255]
Plumbagin	Plumabago zeylanica L	TRAIL	Leukemia	Kasumi-1	GSH↓, ROS↑, DR4/5↑, Caspase8↑	Sensitization	[153]
Plumbagin	Plumabago zeylanica L	BAY-876	Lung	H1299, H1944, H1975, A549	TrxR1↓, O_2_^• −^↑, ROS↑	Sensitization	[164]
Plumbagin	Plumabago zeylanica L	Cisplatin	Tongue	CAL27/CDDP	ROS↑, Mitochondrial superoxide↑, AKT/mTOR↓	Reverse resistance	[256]
Embelin	Embelia ribes	TRAIL	Breast	SUM149	GSH↓, SOD1↓, ROS↑	Sensitization	[154]
Emodin	Rhubarb	Cisplatin	Bladder	T24, J82	GSH↓, ROS↑, MRP1↓	Sensitization	[155]
Emodin	Rhubarb	Platinum	Gallbladder	SGC996	GSH↓, MRP1↓, ROS↑	Sensitization	[257]
Emodin	Rhubarb	Cisplatin	Endometrial	Ishikawa, HEC-IB	NOX↓, P-gp↓, MRP↓, BCRP↓, ROS↓	Sensitization	[172]
Emodin	Rhubarb	Cisplatin	Ovarian	COC1/DDP	ROS↑, GS-X pump↓, MRP1↓	Reverse resistance	[258]
Quercetin	Quercetum	Tamoxifen	Breast	MDA-MB-231, MCF-7	GSH↓, MMP↓	Sensitization	[156]
Quercetin	Quercetum	Cisplatin	Ovarian	SKOV3/CDDP	Trx1/2↓, TrxR1↓, ROS↑	Reverse resistance	[165]
Quercetin	Quercetum	5-FU	Colorectal	HCT116	SOD↓, CAT↓, GPX↓, GR↓, Nrf2/HO-1↓, ROS↓	Reverse resistance	[179]
Quercetin	Quercetum	Cisplatin	Ovarian	SKOV3/CDDP	SOD2↓, CAT↓, GPX1↓, HO-1↓, Nrf2↓	Reverse resistance	[180]
Quercetin	Quercetum	Doxorubicin, Cyclophosphamide	Breast	MDA-MB-231	ROS↓, ERK1/2↓, Caspase3↑	Sensitization	[183]
Artesunate	Artemisia annua	Sunitinib	Renal	KTCTL-26	GPX4↓, GSH↓, ROS↑	Reverse resistance	[259]
Piperlongumine	Piper longum L	Oxaliplatin	Gastric	SGC-7901, BGC-823	TrxR1↓, ROS↑, DNA damage↑, p38/JNK↑	Sensitization	[161]
Piperlongumine	Piper longum L	TRAIL	Breast	MDA-MB-231	ROS↑, p38/JNK↑, DR5↑	Sensitization	[260]
Piperlongumine	Piper longum L	Sorafenib	Liver	HCCLM3, SMMC7721	ROS↑, p-AMPK↑	Sensitization	[261]
Shikonin	Lithospermum erythrorhizon	Gefitinib	Lung	H1650,H1975	TrxR↓, NADPH↓, GSH/GSSG↓, ROS↑, Caspase3↑	Reverse resistance	[163]
Shikonin	Lithospermum erythrorhizon	Cisplatin	Ovarian	A2780/DDP, SKOV3/DDP	GPX4↓, ROS↑, LPO↑	Reverse resistance	[262]
Shikonin	Lithospermum erythrorhizon	Cabazitaxel	Prostate	DU145, PC-3	ROS↑, MMP↓	Sensitization	[263]
Oleanolic acid (OLO-2)	Araliaceae	Cisplatin	Lung	A549/CDDP	TrxR↓, ROS↑, NF-κB↓	Reverse resistance	[166]
Oleanolic acid	Araliaceae	Sorafenib	Liver	Huh7, HepG2	ROS↑, LPO↑	Sensitization	[264]
Sanguinarine	Sanguinaria canadensis	Gefitinib	Lung	H1975	NOX3↑, NADP^+^/NADPH↑, O_2_^• −^↑, H_2_O_2_↑, ROS↑	Reverse resistance	[169]
Sanguinarine	Sanguinaria canadensis	Cisplatin	Ovarian	A2780/R	GSH↓	Reverse resistance	[265]
Ginsenoside Ro	Ginseng	5-FU	Esophageal	ECA-109, TE-1, CT-26	NOX↑, NCF1↑, CYBB↑, ROS↑	Sensitization	[170]
Ginsenoside Rd	Ginseng	Cisplatin	Lung	A549/DDP	Nrf2↓, HO-1↓, NQO1↓, MDR1↓, MRP1↓	Reverse resistance	[177]
Baicalin	Scutellaria baicalensis	5-FU	Gastric	AGS, SGC-7901	NOX1↑, GPX4↓, COX2↑, ROS↑	Sensitization	[118]
Baicalin	Scutellaria baicalensis	Doxorubicin	Breast	MDA-MB-23, MCF-7	Oxidative stress↑, ROS↑, SOD↓, MMP↓	Sensitization	[266]
Ursolic Acid	Rosmarinus officinalis	Oxaliplatin	Colorectal	HCT8, SW480	NADPH↑, ROS↑, P-gp↓, MRP↓, BCRP↓	Sensitization	[171]
Ursolic Acid	Rosmarinus officinalis	Doxorubicin	Breast	MCF-7/ADR	ROS↑, DNA damage↑, p-AMPK↑, p-mTOR↓	Reverse resistance	[267]
Ursolic Acid	Rosmarinus officinalis	Sorafenib	Liver	Hep 3B	ROS↑, SLC7A11↓	Sensitization	[268]
Wogonin	Scutellaria baicalensis	Hydroxycampto- thecin, Cisplatin, Etoposide	Liver	HepG2	Nrf2↓, HO-1↓, NQO1↓, ROS↑, MRP↓	Sensitization	[174]
Wogonin	Scutellaria baicalensis	Doxorubicin	Breast	MCF-7/DOX	Nrf2↓, HO-1↓, NQO1↓	Reverse resistance	[175]
Wogonin	Scutellaria baicalensis	Cisplatin	Head and neck	HN4-cisR, HN9-cisR	Nrf2↓, GSTP1↓, GSH/GSSG↓, ROS↑, p-JNK↑	Reverse resistance	[176]
Wogonin	Scutellaria baicalensis	TRAIL	Prostate	LNCaP	ROS↑, p53↑	Sensitization	[269]
Berberine	Rhizoma coptidis	Lapatinib	Breast	BT-474LapR	Nrf2↓, ROS↑	Reverse resistance	[178]
Berberine	Rhizoma coptidis	Niraparib	Ovarian	A2780, HEY, HO8910	Oxidative stress↑, DNA damage↑	Sensitization	[270]
Parthenolide	Tanacetum parthenium	Mitoxantrone, Doxorubicin	Breast	MDA-MB231	Nrf2↓, CAT↓, MnSOD↓, HSP70↓, ROS↑	Reverse resistance	[181]
Triptolide	Tripterygium wilfordii	Cisplatin	Ovarian	SKOV3PT	ROS↑, NF-κB↓, Caspase3↑	Reverse resistance	[182]
Triptolide	Tripterygium wilfordii	Doxorubicin, Imatinib	Leukemia	HL60/A, K562/G	Nrf2↓, NQO1↓, GSR↓, HO-1↓	Reverse resistance	[271]
Triptolide	Tripterygium wilfordii	Gemcitabine	Bladder	UMUC3, EJ	CAT↓, SOD2↓, ROS↑	Sensitization	[272]
Brusatol	Brucea javanica	Gemcitabine	Pancreatic	PANC-1	Nrf2↓, ROS↑	Sensitization	[273]
Brusatol	Brucea javanica	Trastuzumab	Ovarian, Breast	SK-OV-3, BT-474	Nrf2/HO-1↓, HER2-AKT/ERK1/2↓	Sensitization	[274]
Resveratrol	Berries	5-FU	Colorectal	HT29, SW480, SW620	SOD↑, CAT↓, GPX↓, ROS↑, LPO↑	Sensitization	[184]
Resveratrol	Berries	Platinum	Breast, Choriocarcinoma	MDA-MB-231, JEG-3	O_2_^• −^↑, H_2_O_2_↑, NO_2_^−^↑	Sensitization	[185]
Resveratrol	Berries	Temozolomide	Glioblastoma	SHG44	ROS↑, AMPK↑, mTOR↓, MMP↓	Sensitization	[186]
Resveratrol	Berries	Temozolomide	Glioblastoma	U87MG, GBM8401	ROS↓	Sensitization	[187]
EGCG	Green tea	Gefitinib, Osimertinib	Lung	H1975 (GR)	OXPHOS↑, ROS↑	Reverse resistance	[188]
EGCG	Green tea	TRAIL	Renal	786-O	ROS↑	Sensitization	[189]
EGCG	Green tea	Irinotecan	Colorectal	HCT116,RKO	ER stress↑, ROS↓	Sensitization	[190]
EGCG	Green tea	Doxorubicin	Oral	KB-A-1	Redox↓, Antioxidation↑	Reverse resistance	[191]
Curcumin	Curcuma longa	Docetaxel, Vincristine	Lung	A549/D16, A549/V16	p-ERK↑, p-p38↑, ROS↑	Reverse resistance	[275]
Mitocurcumin	Curcuma longa	Cytarabine	Leukemia	MOLM13	Oxidative stress↑, ROS/p21/CHK1↑, JNK/p38↑	Reverse resistance	[276]
Genistein	Genista tinctoria L	Topotecan	Prostate	LNCaP	ROS↑, Caspase3/9↑	Sensitization	[277]
Genistein	Genista tinctoria L	Arsenic trioxide	Liver	HepG2, Hep3B, SK-Hep-1	ROS↑, Caspase3/9↑	Sensitization	[278]
Gallic Acid	Gallnut	Cisplatin	Lung	H446	ROS↑, MMP↓, p53↑	Sensitization	[279]
Gallic Acid	Gallnut	Paclitaxel	Ovarian	A2780, A2780AD	ROS↑, p-ERK↓	Reverse resistance	[280]
β-Elemene	Curcumae Rhizoma	Cisplatin	Bladder	T24	ROS↑, AMPK↑	Sensitization	[281]
β-Elemene	Curcumae Rhizoma	Cetuximab	Colorectal	HCT116, Lovo	GSH↓, MDA↑, GPX4↓, GLS↓, SLC7A11↓, ROS↑	Sensitization	[223]
Apigenin	Celery	Doxorubicin	Liver	BEL-7402/ADM	Nrf2↓, HO-1↓, ROS↑, PI3K/Akt↓	Reverse resistance	[282]
Saikosaponins	Bupleurum radix	Cisplatin	Cervical, Ovarian, Lung	Hela, Siha, SKOV3, A549	ROS↑	Sensitization	[283]
Rhein	Rhubarb	Oxaliplatin	Pancreatic	PANC-1, MIAPaca-2	ROS↑, MDA↑, PI3K/Akt↓	Sensitization	[284]
Mangostin	Mangosteen tree	Cisplatin	Cervical	HeLa	ROS↑	Sensitization	[285]
Hederagenin	Hedera helix	Cisplatin, Paclitaxel	Lung	H1299, H1975	ROS↑, MAPK↑	Sensitization	[286]
Hederagenin	Hedera helix	Cisplatin	Head and neck	HN3-cisR, HN9-cisR	Nrf2↓, HO-1↓, NQO1↓, xCT↓, GSH↓, ROS↑, p53↑	Reverse resistance	[287]
Tanshinone IIA	Salvia miltiorrhiza	TRAIL	Ovarian	TOV-21G	ROS↑, p-JNK↑, CHOP↑, DR5↑	Sensitization	[288]
Andrographolide	Andrographis paniculate	Cisplatin	Colorectal	HCT116, HT29	ROS↑, MDA↑, ER Stress↑	Sensitization	[289]
Andrographolide	Andrographis paniculate	TRAIL	Prostate	PC-3	ROS↑, p53↑, DR4↑	Sensitization	[290]
Honokiol	Magnolia officinalis	Cabozantinib	Renal	786–0, ACHN	Nrf2↓, HO-1↓, Oxidative stress↑, ROS↑	Sensitization	[291]
Camptothecin	Camptotheca acuminata	TRAIL	Liver	Hep 3B	ROS↑, DR5↑	Sensitization	[292]
Arachidin-1	Peanut	Paclitaxel	Breast	MDA-MB-231	ROS↑, Mitochondrial oxidative stress↑, p53↑	Sensitization	[293]
Glaucocalyxin B	Rabdosia japonica	Cisplatin	Ovarian	A2780, A2780/DDP	ROS↑, p-JNK↑, DNA damage↑	Sensitization	[294]
Alantolactone	Inula helenium	Oxaliplatin	Colorectal	HCT116, RKO	ROS↑, p38↑, p-JNK↑	Sensitization	[295]
Salvianolic acid B	Salvia miltiorrhiza	Vincristine, 5-FU, Cisplatin	Colorectal	HCT8/VCR	ROS↑, P-gp↓, MDR1↓	Reverse resistance	[296]
Gypenosides	Gynostemma pentaphyllum	5-FU	Colorectal	SW-480	ROS↑, DNA damage↑, p53↑	Sensitization	[297]
Dihydroartemisinin	Artemisia annua	Doxorubicin	Cervical	HeLa, SiHa	MDA↑, GSH↓	Sensitization	[298]
Thymoquinone	Nigella sativa	Cisplatin	Oral	SCC-25	ROS↑, LPO↑, DNA damage↑	Sensitization	[299]

## Detoxification effect of NPs: focus on antioxidant properties

The burden of cancer treatment-induced toxicity and adverse effects remains significant, including, but not limited to, various organ toxicities, weight loss, and systemic reactions. Many studies have demonstrated that NPs protect normal cells by attenuating chemotherapy/radiation-induced oxidative stress (Fig. 6C). Another advantage of NPs as adjuncts in cancer therapy is that they do not interfere with the efficacy of existing anticancer agents [192]. Although oxidative stress is one of the anticancer pathways of platinum compounds, it also plays a key role in the systemic toxicities induced by platinum [193]. Honokiol is a natural polyphenolic compound extracted from *Magnolia grandiflora* and has multiple anticancer effects. Systemic application of honokiol prevents cisplatin-induced hearing loss by ROS detoxification without compromising its antitumour effect [192]. Nanoparticulated honokiol attenuates cisplatin-induced nephrotoxicity by maintaining mitochondrial antioxidant capacity [194]. Tanshinone is a natural compound derived from the traditional Chinese medicine *Salvia miltiorrhiza* and has various bioactivities. Tanshinone IIA reduces oxaliplatin-induced neurotoxicity by reducing ROS levels [195]. Curcumin adjuvant administration to cisplatin therapy exerts antioxidant and protective effects by upregulating the endogenous antioxidant defense system (Nrf-2/HO-1) in cochlear cells [196]. The combination of curcumin and α-tocopherol protects against cisplatin-induced hepatotoxicity by reducing NOX and oxidative markers (ROS and MDA) and increasing CAT [197]. In addition, curcumin pretreatment significantly increases Nrf2 in the nucleus and decreases the expression of Keap1, as well as reverses the doxorubicin-induced reduction of HO-1 and NQO1, which provides a rational mechanism against doxorubicin-induced neurotoxicity [198]. Mitochondrial dysfunction-induced oxidative stress represents a major cause of doxorubicin-induced cardiotoxicity. Oridonin shows a cardiac protective effect and improves doxorubicin-induced oxidative stress. The pretreatment with oridonin has been shown to significantly elevate the activities of antioxidant defense components such as SOD, Mn-SOD, HO-1, and GPX, while reducing NOX subunit p47phox activation levels [199]. Another benefit of combining oridonin with chemotherapeutic agents is the reduction in the doses of chemodrugs, which serves to decrease the incidence of adverse effects [200]. Similarly, berberine alleviates doxorubicin-induced cardiotoxicity by suppressing oxidative stress via activating the Nrf2 pathway [201]. A recent study has demonstrated that kaempferol offers protection against doxorubicin-induced nephrotoxicity by suppressing ROS/ASK1-mediated MAPK signaling and attenuating oxidative stress [202]. Previous reviews have explored phytochemicals with radioprotective effects, primarily including phenols, flavonoids, and polysaccharides [203]. For example, ferulic acid pretreatment has been shown to protect peripheral blood mononuclear cells from radiation damage by significantly preventing radiation-induced ROS generation, restoring GSH, and increasing NF-κB and Nrf2 levels within the nuclei [204]. The testis, as one of the most radiosensitive organs, can be significantly impaired by even low doses of radiation. Kim et al. reported that genistein has a protective effect against radiation-induced testicular injury, primarily through the mitigation of ROS [205]. Overall, the anti-oxidative pathway may serve as a major pathway of NPs in normal cells within the context of chemotherapy/radiation protection.

## Contemporary research: potential challenges and promising future

**Fig. 7:**
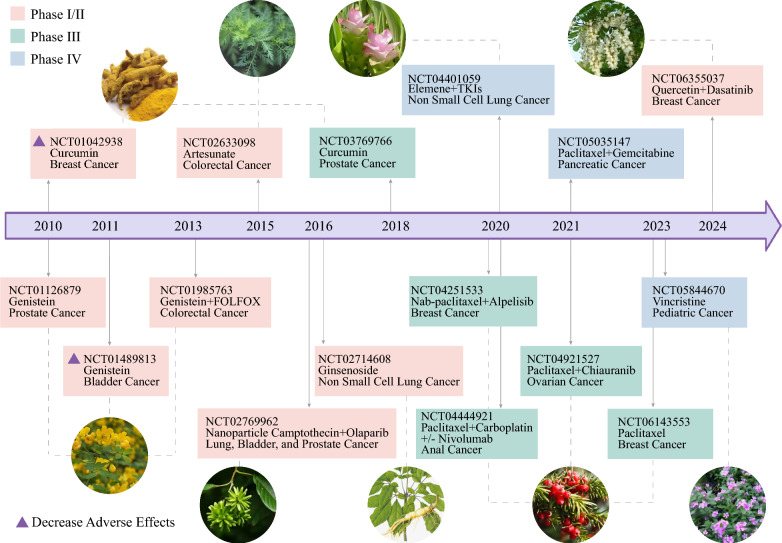
Advances in clinical trials of anticancer drugs based on NPs over the past few decades. Clinical trials of anticancer drugs based on NPs are emerging with maturity, whether they are used alone, in combination with sensitizers, or to reduce adverse effects, indicating promising prospects for the benefit of patients

Although NPs are widely available and affordable, they still face challenges from the laboratory to the clinic due to limitations in their properties, effectiveness, safety, and translation. To date, numerous improvements in NP-based properties, derivative preparations, and novel delivery systems have been widely reported as advanced approaches to address these challenges and increase their efficiency. With progress in product conversion and clinical trials (Fig. 7), NPs are well developed and advancing, providing promising prospects for cancer treatment strategies.

### Overcoming limitations: novel redox-sensitive materials

**Fig. 8:**
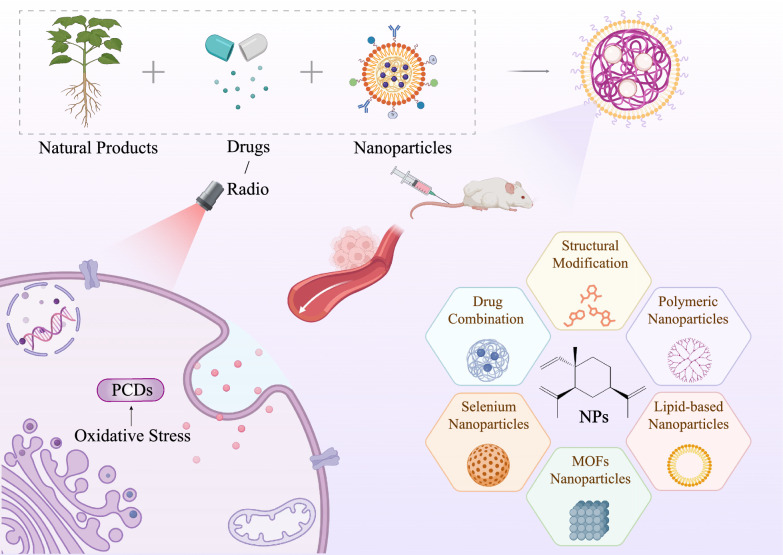
Novel redox-sensitive materials enhance the utilization efficiency of NPs. Considering the abnormal redox microenvironment in cancer cells, novel redox-sensitive materials (structural modifications, derivatives, and nanodelivery systems) based on NPs and drugs serve as innovative approaches to overcome the limitations of NPs and may further induce cascade amplification of oxidative stress to exert significant anticancer activity

Most NPs have common characteristics such as poor solubility, low stability, and low bioavailability [206]. An innovative approach to overcome these limitations is the multifunctional design of structural modifications and drug delivery systems based on the structure‒activity relationship (SAR). For example, the structural modification of neohesperidin using an immobilized lipase improves lipophilicity, benefiting its action on the cancer cell wall and enhancing bioavailability in vivo [207]. The water solubility of β-elemene could be improved by introducing hydrophilic moieties like hydroxyl and amino groups [208]. A novel nanodrug delivery system was constructed to coload paclitaxel and triptolide, with favourable stability and biocompatibility, enhancing ferroptosis in lung cancer cells via ROS accumulation [209]. Zhen et al. prepared biomineralized oxidized EGCG-molybdenum ion-coordinated nanoparticles with good solubility and long-term safety that reacted with GSH via Michael addition to form aggregates, which further inhibited cancer progression [210]. Yan et al. prepared a nano-metal-organic framework (MOF)-encapsulated dihydroartemisinin, with better cytocompatibility and anticancer activity, and induced apoptosis in ovarian cancer cells by inhibiting ROS [211]. Cui et al. synthesized hawthorn fruit extract-mediated selenium nanoparticles, which were stable and inhibited liver cancer cells by inducing oxidative stress and mitochondrial dysfunction [212]. Collectively, these novel redox-sensitive materials may induce a cascade amplification of oxidative stress and exert significant anticancer activity (Fig. 8).

### Preclinical strategies: improving selectivity and evaluating safety

Although NPs can attenuate the adverse effects of conventional therapies, we cannot hold the preconceived notion that they are absolutely safe. Several approaches could avoid undesirable effects and achieve the "high efficacy and low toxicity" of NPs by increasing selectivity and reducing off-target effects. The cisplatin and curcumin coloaded liposome system extends the drug duration and promotes drug accumulation in tumours. The nano-liposomes generate more ROS compared with the single drug agent [213]. Liposomes loaded with paclitaxel-doxorubicin respond to stimulation of the tumour microenvironment with high levels of ROS/GSH to release drugs [214]. Advanced formulations of resveratrol increase the accumulation of loaded drugs in cancer areas and prevent off-target drug release [215]. A ROS-responsive artesunate complex carrier was prepared, with hyaluronic acid in this carrier enabling deep tumour penetration and selective drug release [216]. In addition, some biological probes based on NPs have been prepared and can be used for target identification and improving selectivity. Efforts are needed to evaluate the safety of NPs in vitro and in vivo before any pharmaceutical administration, with a focus on distribution, metabolism, and organ toxicity. The clinical application of celastrol is limited due to severe side effects. Compound 19–048 derived from celastrol significantly reduces side effects according to target identification and structural modifications [63]. The identification, response, and management of potential toxicity and side effects are critical challenges for the clinical application of NPs, which still have a long way to go.

### Product conversion: technology and science

The transformation of achievements and standardized production is an important step for bringing a natural compound from the laboratory to the clinic. Emerging technologies and advances in science provide opportunities for the discovery, production, and engineering of NPs. Traditional NP-based research starts with screening, identifying, and extracting, which is a costly process. Recently, analytical techniques, genome mining and engineering, and microbial culturing have been identified as advanced technologies for discovering NPs [14]. Most NPs require improvements before they can be used in the clinic; rather than being used directly, biotechnology and engineering will be highly important in this context. Plant cell suspension culture is emerging as a viable technology to overcome quality and quantity challenges during the production of NPs [217]. Li et al. reported the use of protein engineering for improving and diversifying NP synthesis [218]. Additional challenges with production that need to be resolved include purity and titer, quality control, standardization, and sustainability. Science and technology progress in other important research areas, such as computational tools [219], database platforms [220], and multiomics [221], also significantly influence NP-based therapies and offer great potential for the development of NPs.

### Promising progression: clinical trials and patents

Given the promising aspects of anticancer therapy, several NPs and their analogs or derivatives are emerging for use in clinical trials or patents (Table 3). Elemene, a major ingredient of the traditional Chinese medicine *Curcumae Rhizoma*, has been confirmed by our team to have inhibitory activity against various cancers [222, 223]. We have disclosed several patents for elemene oral microemulsion (US20120322892A1), injectable solution (CN101306181A), and sustained-release tablets (US20130059922A1). The synergistic treatment of elemene plus TKIs for EGFR-mutated advanced non-small-cell lung cancer has entered Phase IV clinical trials (NCT04401059). Clinical trials of genistein in combination therapy have been conducted in breast cancer (NCT00244933) and lung cancer (NCT01628471). Recently, quercetin combined with dasatinib has been approved to enter Phase II clinical trials (NCT06355037) for reversing chemotherapy resistance in breast cancer. In addition, the anticancer applications of some classic NPs such as paclitaxel, vincristine, and camptothecin are already becoming increasingly mature. Collectively, the application of NPs into clinical practice is a tantalizing prospect that may facilitate patient benefits.

**Table 3:** Advances in clinical trials of anticancer drugs based on NPs have occurred over the past few decades

Posted	Identifiers	NPs-interventions	Conditions	Phase
2005	NCT00244933	Genistein + Gemcitabine	Breast Cancer	II
2005	NCT00256334	Resveratrol	Colon Cancer	I
2006	NCT00376948	Genistein + Gemcitabine + Erlotinib	Pancreatic Cancer	II
2008	NCT00764036	Artesunate	Metastatic Breast Cancer	I
2010	NCT01042938	Curcumin C3 Complex	Breast Cancer	II
2010	NCT01126879	Genistein	Prostate Cancer	II
2011	NCT01333917	Curcumin C3	Colorectal Cancer	I
2011	NCT01490996	Curcumin + FOLFOX	Colonic Cancer	I/II
2011	NCT01489813	Genistein	Bladder Cancer	II
2012	NCT01628471	Genistein + Decitabine	Non Small Cell Lung Cancer	I/II
2013	NCT01985763	Genistein + FOLFOX/FOLFOX-Avastin	Colorectal Cancer	I/II
2015	NCT02633098	Artesunate	Colorectal Cancer	II
2015	NCT02499861	Genistein + Decitabine	Solid Tumors, Leukemia	I/II
2016	NCT02714608	Ginsenoside H dripping pills	Non Small Cell Lung Cancer	II
2016	NCT02769962	Nanoparticle Camptothecin + Olaparib	Lung, Bladder, and Prostate Cancer	I/II
2018	NCT03769766	Curcumin	Prostate Cancer	III
2019	NCT03980509	Curcumin	Breast Cancer	I
2020	NCT04401059	Elemene + EGFR-TKIs	Non Small Cell Lung Cancer	IV
2020	NCT04444921	Paclitaxel + Carboplatin + Nivolumab	Anal Cancer	III
2020	NCT04251533	Nab-paclitaxel + Alpelisib	Triple Negative Breast Cancer	III
2021	NCT05035147	Albumin-bound paclitaxel	Pancreatic Cancer	IV
2021	NCT04921527	Paclitaxel + Chiauranib	Ovarian Cancer	III
2022	NCT05456022	Quercetin	Oral Cancer	II
2023	NCT05844670	Vincristine	Pediatric Cancer	IV
2023	NCT05747313	Vincristine + Chidamide	Triple Negative Breast Cancer	I/II
2023	NCT06143553	Paclitaxel Polymeric Micelles	Metastatic Breast Cancer	III
2024	NCT06355037	Quercetin + Dasatinib	Triple Negative Breast Cancer	II

## Conclusions and perspectives

Oxidative stress is a “double-edged sword” in cancer biology and treatment. Although the antioxidant capacity of NPs endows them with the ability to favourably modulate some pathological processes and reduce the side effects of chemotherapeutic drugs, caution is warranted when discussing the mechanisms and the use of NPs as sensitizers. In contrast to their antioxidant activity, the synergistic effect of NPs, which is mediated by increasing oxidative stress, has been documented in many cancers, and such a phenomenon is not uncommon in anticancer drugs. A reasonable explanation for this paradoxical phenomenon is that an antioxidant in one system is not necessarily an antioxidant in all systems. NPs may exert distinct effects on the regulation of redox reactions in different types of cells, i.e., nontumour cells undergoing stressful conditions versus cancer cells. In the former, NPs exert a protective effect as antioxidants and detoxifiers, whereas in cancer cells, NPs are mostly converted to mediate pro-oxidant activity, promoting cell death and overcoming therapeutic resistance. In other words, NPs appear to be flexible and display relatively selective benefits in different types of cells. Several mechanisms seem to explain this phenomenon on the basis of the preceding discussion.

The heterogeneity in the redox state between cancer and normal cells provides a biological basis for regulating redox reactions as a strategy to selectively kill cancer cells with NPs. It has been well-established that cancer cells exhibit higher levels of oxidative stress compared with normal cells. Excessive oxidative stress not only is an inducing factor for carcinogenesis but also renders cancer cells more vulnerable to oxidative damage because they survive with a heightened basal level of ROS-mediated signalling, which is required for high metabolism, enhanced energy, and rapid proliferation [13, 224, 225]. Under these conditions, further exposure to threshold-excessive ROS overwhelmingly pushes cancer cells toward peroxidative damage and cell death. In contrast, normal cells may tolerate exogenous intervention better because of their low baseline levels of oxidant signalling and normal metabolic modulation [224, 225]. For example, flavonoids can scavenge low levels of ROS in normal cells. However, high and sensitive levels of ROS in cancer cells are difficult to scavenge with flavonoids. Furthermore, the mitochondrial membrane disruption and decrease in the membrane potential caused by flavonoids are accompanied by a pro-oxidative response [226]. In addition, considering that part of the anticancer mechanism of NPs occurs through exofacial protein modification, the extracellular redox state may also contribute to this selective effect. There is evidence that in contrast to the oxidative extracellular redox state of normal cells, cancer cells exist in a reduced extracellular environment [227]. For example, it is possible that the exofacial thiol targets of parthenolide are reduced on cancer cells and, thus, able to interact with parthenolide but are oxidized on normal cells and, thus, unavailable for interaction with parthenolide [77].

Another possibility links the different effects of NPs to redox-related targets from the perspective of molecular biology. For example, the antioxidant and pro-oxidant effects of quercetin depend on the availability of reduced intracellular GSH. In the presence of high GSH concentrations in normal cells, the reversibility between oxidized quercetin and GSH ensures protection against quercetin toxicity [228]. Parthenolide selectively activates NOX and increases oxidative stress in prostate cancer cells; however, parthenolide may become trapped in normal cells by sulfa-Michael addition under the catalysis of GSTs, rendering them more tolerant [79, 229]. Xu et al. identified Keap1 as a determinant controlling the differential effects of parthenolide on cancer and normal cells [230]. Parthenolide susceptibility is also determined by CAT activity [231]. In summary, this speculation is plausible, and we hypothesize that the antioxidant mechanism of NPs in normal cells may be accessed by stimulating the production of more antioxidants, following a "consumption–replenishment" model. However, this concept seems insufficient to explain all cases because some cancer cells, as mentioned before, particularly drug-resistant cells, also exhibit significantly increased antioxidant defenses. For these cells, targeting the antioxidant system is a strong strategy for sensitization and reversing resistance.

Importantly, the anticancer activity of the NPs is not immutable, with several pieces of evidence indicating that the anticancer effects of the NPs involve both their pro-oxidant and antioxidant activities. Specifically, the effects of NPs may be influenced by different concentrations, durations, test conditions, and cancer types, which may explain their biphasic effects. As mentioned previously, quercetin at low concentrations has antioxidant activity, whereas high concentrations of it synergize to increase the production of ROS [111]. Another hypothesis for the biphasic effect is that a burst of ROS is not always beneficial for inducing cytotoxicity-PCD but may also mediate protective autophagy in cancer cells. For example, ROS-mediated autophagy induction by honokiol in prostate cancer cells is cytoprotective [232]. In this context, the use of antioxidants is promising. As reported by Lin et al., the combination of resveratrol and temozolomide affects malignant glioma by inhibiting ROS/ERK-mediated autophagy [187].

In summary, it has been widely confirmed that NPs serve as adjuvants for enhancing drug sensitivity and overcoming resistance, as well as for reducing adverse effects by regulating the redox microenvironment. NPs exhibit differential, selective, and bidirectional effects on tumour redox states. The underlying mechanisms of this phenomenon appear complex, multifaceted, and interconnected, involving different cellular contexts, molecular regulations, pathological landscapes, and conditions of action. Whether there are critical thresholds between these effects and how to identify and delineate them to ensure the optimal effect of NPs as intended in different diseases remain unresolved issues. Future research is expected to explore more specific targets and mechanisms of NPs, critically identifying the roles of the targets in redox balance and the differences between normal and malignant environments. Currently, research progress on novel redox-sensitive NP materials is inspiring, with some NPs already being developed for clinical trials. These findings support the promising prospects of NPs, which are expected to provide clinical and patient benefits in the future.

## Data Availability

Not applicable.

## Abbreviations

NPs: Natural products
ROS: Reactive oxygen species
RNS: Reactive nitrogen species
H_2_O_2_: Hydrogen peroxide
NO: Nitric oxide
GSH: Glutathione
GSSG: Glutathione disulfide
Trx: Thioredoxin
TrxR: Thioredoxin reductase
NADPH: Nicotinamide adenine dinucleotide phosphate
OXPHOX: Oxidative phosphorylation
NOX: NADPH oxidase
LOX: Lipoxygenase
COX: Cyclooxygenase
NOS: Nitric oxide synthase
GPX: Glutathione peroxidase
Grx: Glutaredoxin
GST: Glutathione S-transferase
NAC: N-acetylcysteine
Prx: Peroxiredoxin
NF-κB: Nuclear factor kappa B
Nrf2: Nuclear factor erythroid 2-related factor 2
Ref-1: Redox factor-1
TXNIP: Thioredoxin-interacting protein
CAT: Catalase
SOD: Superoxide dismutase
HO-1: Haem oxygenase-1
PCD: Programmed cell death
GLS: Glutaminase
ER: Endoplasmic reticulum
NQO1: NAD(P)H dehydrogenase [quinone] 1
EGCG: Epigallocatechin-3-gallate
Cys: Cysteine
Sec: Selenocysteine
LPO: Lipid peroxidation
5-FU: 5-Fluorouracil
TRAIL: Tumour necrosis factor-related apoptosis-inducing ligand
EGFR: Epidermal growth factor receptor
TKI: Tyrosine kinase inhibitor
MOF: Metal–organic framework
